# SNR‐Efficient Inhomogeneous Magnetization Transfer (ihMT) for Clinical Applications at 7 T

**DOI:** 10.1002/mrm.70419

**Published:** 2026-05-11

**Authors:** Timothy Anderson, Niklas Wallstein, Lucas Soustelle, Thomas Troalen, Gopal Varma, David C. Alsop, Maxime Guye, Guillaume Duhamel, Olivier M. Girard

**Affiliations:** ^1^ Aix Marseille Univ, CNRS, CRMBM Marseille France; ^2^ APHM Hôpital Universitaire Timone, CEMEREM Marseille France; ^3^ Siemens Healthcare SAS Courbevoie France; ^4^ Radiology, Division of MR Research, Beth Israel Deaconess Medical Center Harvard Medical School Boston Massachusetts USA

**Keywords:** inhomogenous magnetization transfer $B_{1}^{+}$ correction, high resolution, myelin, neuroimaging, UHF

## Abstract

**Purpose:**

Inhomogeneous MT (ihMT) is an MRI modality sensitive to Myelin, the lipid‐rich membrane surrounding the axons. A novel ihMT saturation strategy and its associated $B_{1}^{+}$ correction at UHF are proposed. The aim is to generate a strong ihMT effect within a clinically compatible scan time while complying with SAR constraints at 7 T.

**Methods:**

The MT preparation module of the proposed approach was shortened, reducing power deposition and allowing for shorter scan time while maximizing SNR efficiency. Optimization of the sequence was validated on N=4 (2 mm iso.) on a 7 T clinical scanner. Example high resolution images were acquired (1–1.4 mm iso.). Model‐based $B_{1}^{+}$ corrections were applied and their impact on ihMT quantification was studied. Numerical simulations and error quantifications were performed to complement experimental data.

**Results:**

Fewer bursts ($N_{B}=5$) of cosine‐modulated MT preparation pulses ($N_{P}=1$) performed at maximal $B_{1}^{\mathrm{rms}}$ yielded strong ihMT signal with better SNR efficiency as compared to previously proposed long saturation trains ($N_{B}=10$). $B_{1}^{+}$ correction showed a reliability range ($B_{1,\mathrm{rel}}^{+}\in \left[80,120\right]\%$) which challenged applicability in strong hypo‐intense $B_{1}^{+}$ areas. High resolution acquisitions showed acceptable SNR (≥25) for resolutions down to 1 mm iso., enabling studies of fine structures (e.g., deep gray nuclei) with added specificity provided by ihMT.

**Conclusion:**

ihMT applications at 7 T are enabled. While whole‐brain imaging requires further work to compensate for $B_{1}^{+}$ inhomogeneities, localized applications (e.g., Thalamus) are immediately feasible within clinically compatible scan time (< 8 min). Similar sequence improvements are expected at lower field strengths (e.g., $B_{0}=3\;T$).

## 1 Introduction

Inhomogeneous MT (ihMT) [1, 2, 3] is an MRI modality sensitive to Myelin [4], the lipid‐rich membrane surrounding the axons. IhMT provides a probe of interest for clinical research in neuroimaging as myelin changes are characteristic to many central nervous system pathologies (e.g., amyotrophic lateral sclerosis [5], multiple sclerosis [6, 7]).

Over the last decade, clinical MRI has delved deeper into Ultra‐High Field (UHF, $B_{0}\geq 7\;T$) [8], which, among others, allows for probing higher resolutions due to increased Signal‐to‐Noise Ratio (SNR) from higher spin polarization and faster spin precession.

However, as an RF saturation based technique, ihMT is limited by Specific Absorption Rate (SAR) [9]. SAR typically scaling as the power integral of static magnetic field ($B_{0}$) amplitude [10], this constraint is usually exceeded much faster at UHF than lower field strengths. In addition, because of RF propagation effects and associated interference patterns, the local SAR (defined as the average over 10 g) is the primary limiting factor at UHF, instead of the global (head or exposed‐body) SAR [11]. This is partially remediated by replacing the usual volume body coil with proximal excitation coils in UHF systems. One consideration remains: local SAR limits, monitored over two time‐scales: 10 s and 6 min time‐average sliding windows. MRI sequences must abide by both in order to be run safely on a clinical system, which can come at the cost of a higher total acquisition time (TA) to reduce average power deposition. In clinical applications however, longer sequences are impractical at best and unusable at worst (e.g., from lack of patient comfort, exacerbated motion artifacts [12]), and UHF ihMT sequences run the risk of being far too long to compensate for the substantial increase in power deposition.

Another challenge with UHF, is the characteristic higher transmit field ($B_{1}^{+}$) inhomogeneity [13] compared to those occurring at lower field strengths (≤ 3 T), which may render ihMT sequences challenging in peripheral regions of the brain with strong $B_{1}^{+}$ hypo‐intensities (e.g., temporal lobes and cerebellum) [14]. In practice, the average dispersion of the $B_{1}^{+}$ field can reach 25% (relative standard deviation) of the nominal value at UHF over a typical human brain using a single‐channel transmission coil (e.g., 1‐Tx/32‐Rx head coil, Nova Medical, Wilmington, USA), which is larger than the ˜11% relative STDev usually found at 3 T using body‐coils. Suitable $B_{1}^{+}$ correction strategies to allow for viable ihMT imaging at UHF are thus required.

**FIGURE 1:**
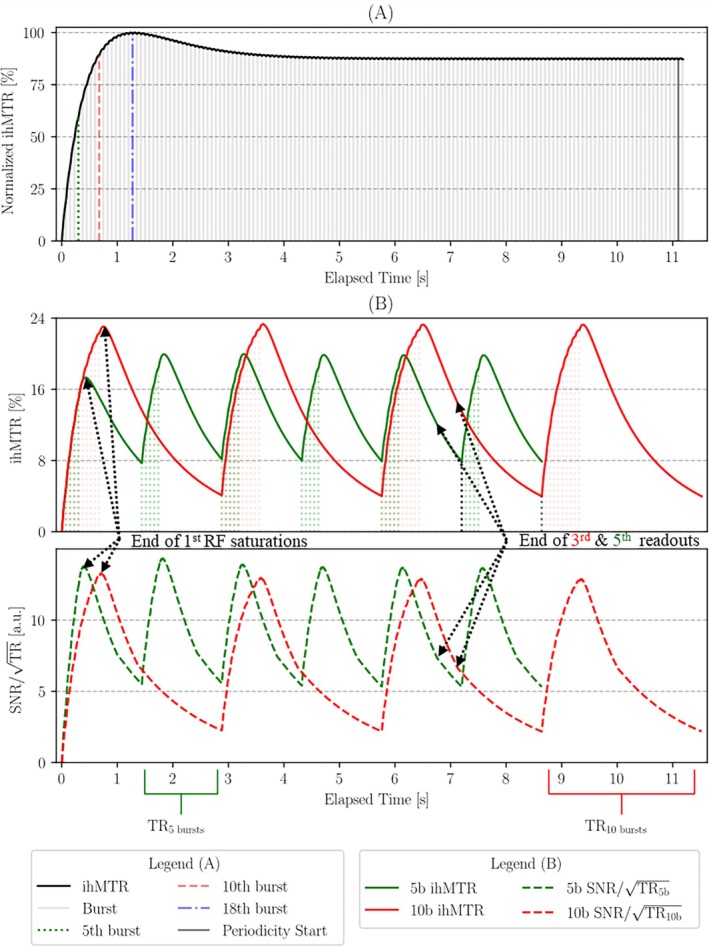
(A) Simulation of the ihMTR evolution for a unique cosine‐modulated RF saturation module parametrization repeated until a periodic state is achieved in the system. (B) Comparison of ihMTR (top) and $\mathrm{SNR}/\sqrt{\mathrm{TR}}$ (bottom) evolutions over multiple TRs for 2 cosine‐modulated sequences parametrizations with equal power deposition: 5 bursts using TR=1440 ms, and 10 bursts using TR=2880 ms. See simulation details in *Material and Methods*.

In this work we propose a novel ihMT saturation strategy and its associated $B_{1}^{+}$ correction [15], for use in myelin imaging at UHF thus improving over previous state‐of‐the‐art ihMT saturations [16, 17, 18, 19] which are incompatible with UHF MRI clinical constraints. The novelty of the proposed approach is based on making the most efficient use of an MT preparation module and its associated rapid Gradient Echo (RAGE) readout module to generate a strong ihMT effect within a clinically compatible scan time while complying with the SAR constraints at 7 T. The key principle of the proposed approach is that RAGE pre‐saturation modules do not need to reach a pseudo‐steady saturation state (defined as a periodic magnetization behavior from one saturation pulse burst to the next) and may be shortened substantially (Figure 1). Shorter pre‐saturation modules lead to lower power depositions which in turn allow for shorter sequence TRs and shorter TA, under SAR constraints. Of note, despite a lower maximum amplitude of the ihMT ratio (ihMTR) metric, the SNR efficiency (defined as $\mathrm{SNR}/\sqrt{\mathrm{TR}}$) may be increased using shorter saturation modules (Figure 1B). The proposed strategy is referred hereafter as “SNR‐Efficient saturation.”

This paper will cover the optimization of SNR‐Efficient saturation along with the assessment of the robustness of the associated model‐based $B_{1}^{+}$ inhomogeneity correction method optimized for white matter (WM) but applied everywhere. As a first step, an iterative procedure between experiments and biophysical model fitting was applied to determine the optimal SNR‐Efficient saturation configuration (i.e., the optimal sequence RF saturation variables) and derive $B_{1}^{+}$ dependencies of the ihMT signal for subsequent corrections. Then, the optimal SNR‐Efficient configuration was applied experimentally to assess the $B_{1}^{+}$ inhomogeneity correction efficacy in WM and to acquire high resolution ihMT images of the human brain.

## 2 Methods

### 2.1 Materials

Experiments were performed on a 7 T clinical scanner (MAGNETOM Terra, Syngo Software version VE12U‐SP01, Siemens Healthineers, Forchheim, Germany) using a 32‐channels Rx, single Tx Nova coil run under the manufacturer's first level controlled operating mode. Written informed consent was obtained from all participants, according to general guidelines and institutional ethical committee approval. Specifications of all software used for processing are available in Supporting Information (Section S1).

### 2.2 Methods

The MR study was separated into two sets of experiments: Experiment A to fit biophysical parameters of the selected ihMT model from experimental data, which was then used to derive an SNR‐Efficient saturation sequence and an $\text{ihMTR}=f\left(B_{1}^{+}\right)$ correction. It was performed on N=4 healthy volunteers (3:1 male:female; mean age of 31 ± 8 years), over 2 sessions (“test–retest”), consisting of exiting and reentering the MRI scanner after a break of at most 30 min. Experiment B for $B_{1}^{+}$ correction validation and high resolution ihMT imaging. It was performed over 1 session on one healthy volunteer as a feasibility and validation study (female; 30 years old; independent from volunteers of Experiment A).

#### 2.2.1 Image Acquisition

For each volunteer, an anatomical reference image was acquired in sagittal orientation using an MP2RAGE [20] sequence as described in Massire et al. [21] during the first session (TR=5000 ms, ${\mathrm{TI}}_{1}=900$ ms, $FA_{1}=6$°, ${\mathrm{TI}}_{2}=2750$ ms, $FA_{2}=5$°, TE=3.13 ms, ES=7.4 ms, readout bandwidth bdw=240 Hz/px, $N_{\mathrm{ADC}}=192$, GRAPPA 3x1 acceleration, 6/8 phase and partition partial Fourier). A transmit field ($B_{1}^{+}$) map was acquired in every session (product pre‐saturated turbo‐FLASH sagittal interleaved and 2D multi‐slice $B_{1}^{+}$ mapping sequence, with a target flip angle of 90° and a matching FOV) [22].

**FIGURE 2:**
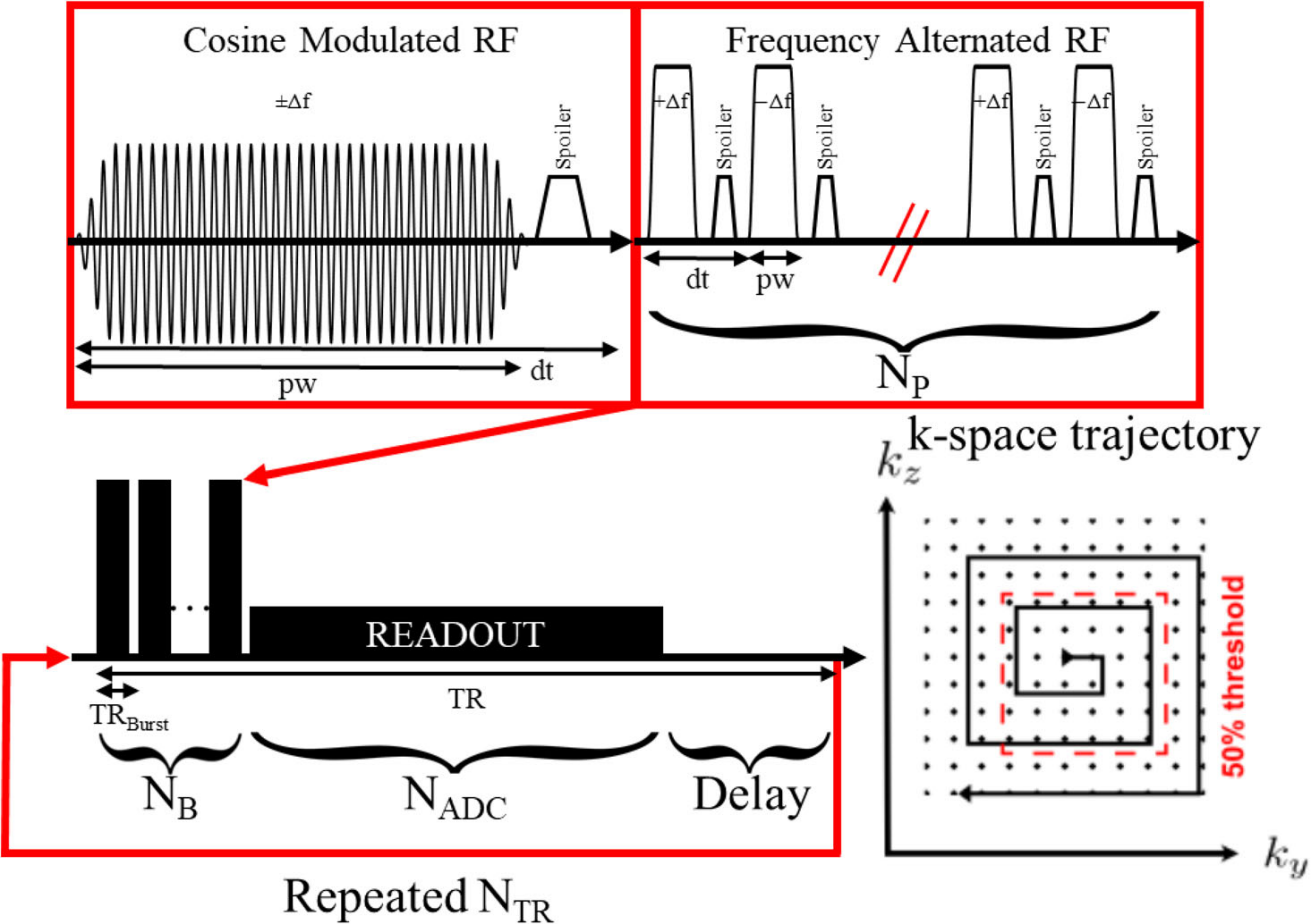
Illustration of dual‐offset saturation schemes in a single TR of the ihMT experiment and the k‐space trajectory for acquisition. For cosine‐modulated dual saturation a single pulse (width pw) is used (top left), whereas for frequency‐alternated, a train of $N_{P}$ pulses separated by spoiler gradients is used (top right). Each MT burst (tall, black‐filled rectangle) is made up of Tukey‐shaped RF spaced by spoiling gradients. The number of pulses and associated duration is varied depending on the way the dual offset saturation is performed. Each delay between $N_{B}$ bursts has spoiling gradients (bottom left). The readout module encodes the signal (view ordering) in a centric‐out cartesian spiral trajectory. TR finishes with a recovery delay to lower power deposition for the 10 s SAR constraint. MT RF pulses are turned off to lower power deposition for the 6 min SAR constraint when 50% of the k‐space has been filled [23].

An MT‐prepared RAGE prototype sequence with a centric‐out cartesian spiral trajectory [23] (Figure 2, bottom right) was used for ihMT acquisitions. The trajectory was acquired as follows: phase encoded k‐space lines ($k_{y}$, $k_{z}$) were sorted from increasing radial distance from the center and acquired sequentially in that order. The central half of these phase encoded k‐space lines were acquired first, then MT preparation was turned off for the remaining half. The MT preparation consisted of repetitions of $N_{B}$ bursts of $N_{P}$ RF saturation pulses played at fixed peak $B_{1}^{+}$ amplitude. Bursts were repeated every $TR_{\text{Burst}}$, for a root‐mean squared burst amplitude $B_{1,\text{Burst}}^{+,\mathrm{rms}}$ for the MT RF pulses. $N_{P}$ and pulse duration (pw) varied based on whether a cosine‐modulated (CM) or frequency‐alternated (ALT) approach was used for dual‐offset saturation (Figure 2, top left and right). Dual offset CM pulses can be long or short because they irradiate both positive and negative offsets within a single pulse. The ALT approach required shorter pulses to achieve a relatively fast frequency‐offset switching over consecutive pulses (millisecond scale). MT pulse specifications of the different configurations were based on previous studies optimized for lower field strengths [18, 24]. Specifically, all sequences with CM for dual‐offset saturation used a single Tukey pulse per burst [24, 25]: offset frequency $\Delta f^{\pm }=\pm 7$ kHz, Tukey shape factor r=0.2, pulse width pw=5 ms, dual‐offset peak CM pulse amplitude $B_{1,\text{Pulse}}^{+,\text{peak dual}}=23$ μT, single‐offset peak pulse amplitude $B_{1,\text{Pulse}}^{+,\text{peak single}}=16$ μT, and root‐mean squared pulse amplitude $B_{1,\text{Pulse}}^{+,\mathrm{rms}}=15$ μT for both single‐ and dual‐sided off‐resonance saturations. In contrast, all sequences that used ALT for dual‐offset saturation had an even number of RF Tukey pulses per burst (with offset frequencies alternating between $\Delta f^{+}=7$ kHz and $\Delta f^{-}=-7$ kHz every pulse), Tukey shape factor r=0.3, pw=1 ms, inter‐pulse period dt=1.5 ms, $B_{1,\text{Pulse}}^{+,\text{peak}}=23$ μT, and $B_{1,\text{Pulse}}^{+,\mathrm{rms}}=21$ μT. A $B_{1}^{+,\text{peak}}\leq 23$ μT limit was imposed by hardware constraints associated with the RF transmission coil. Readout modules were the same for all sequences, with bdw=240 Hz/px, $N_{\mathrm{ADC}}=88$ readouts per TR, readout pulse flip angle FA=5°, TE=3.6 ms, ES=7.9 ms, and GRAPPA 3x1 acceleration. All sequences also used a 50% Partial Fourier Saturation scheme [23].

All ihMT sequences acquired five volumes sequentially: $MT_{0}$, $MT_{+}$, $MT_{\pm }$, $MT_{-}$, and $MT_{\mp }$, where $MT_{0}$ corresponds to signals obtained with no RF saturation, $MT_{+}$ and $MT_{-}$ correspond to signals obtained with single sided RF saturation with positive and negative offset, respectively, and $MT_{\pm }$ and $MT_{\mp }$ correspond to signals obtained with dual‐offset RF saturation.

All volumes were acquired with at least 5 s of dummy repetitions. MT pulses were turned off after acquiring 50% of the phase encoding k‐space lines (Figure 2, bottom right). This strategy improved the overall SAR tradeoff and provided better compromise between $B_{1}^{+,\text{peak}}$ and total average power. This is particularly interesting since ihMT signals typically show quadratic or super‐quadratic growth with RF amplitude, especially in low $B_{1}^{+}$ conditions [26]. Adjustments of $N_{P}$, $N_{B}$, and ${\mathrm{TR}}_{\text{burst}}$ values were part of the SNR‐Efficient saturation module optimization (Experiment A). Specifications of the acquired sequences are available in Table 1. A sequence chronogram is available in Supporting Information (Section S2).

**TABLE 1:** Parameters of the SNR‐efficient ihMT sequences for Experiment A (top) and Experiment B (bottom).

Experiment A	MT module	Bursts ($N_{B}$)	Pulses per burst ($N_{P}$)	TA acquired [min:s]	$TR_{\text{RAGE}}$ acquired/advised [ms]	$TR_{\text{Burst}}$acquired/advised [ms]	Figures
Session 1	**CM 5B1P/** $\theta_{\mathrm{ref}}^{\mathrm{CM}}$	**5**	**1**	**5:47**	**1440/1450**	**75/145 [75–230]**	**1, 4, 5, 6**
	ALT 3B2P	3	2	3:32	840/920	70/110 [50–220]	4, 5, 6
	ALT 3B4P	3	4	5:14	1300/1290	85/175 [85–325]	5
	ALT 3B6P	3	6	7:42	1960/1930	95/195 [100–375]	4, 5, 6
	**ALT 4B4P/** $\theta_{\mathrm{ref}}^{\mathrm{ALT}}$	**4**	**4**	**6:46**	**1720/1720**	**85/165 [85**–**310]**	**4, 5, 6**
Session 2	CM 3B1P	3	1	3:47	903/953	75/125 [65–235]	4, 5, 6
	CM 4B1P	4	1	4:53	1190/1160	75/150 [75–225]	5
	**CM 5B1P/** $\theta_{\mathrm{ref}}^{\mathrm{CM}}$	**5**	**1**	**5:47**	**1440/1450**	**75/145 [75–230]**	**1, 4, 5, 6**
	CM 6B1P	6	1	6:46	1720/1730	70/135 [70–230]	5
	CM 7B1P	7	1	7:59	2030/2020	70/130 [70–230]	4, 5, 6
	**ALT 4B4P/** $\theta_{\mathrm{ref}}^{\mathrm{ALT}}$	**4**	**4**	**6:46**	**1720/1720**	**85/165 [85–310]**	**4, 5, 6**

Experiment B	MT module	Resolution	Acquisition matrix	TA [min:s]	Figures
**Variable** $V_{\mathrm{ref}}$	**CM 5B1P/** $\theta_{\mathrm{ref}}^{\mathrm{CM}}$	**2.0 mm iso**.	**88 × 128 × 80**	**5:47**	**1, 6, 7, 8**
Whole Brain High Resolution	CM 5B1P/$\theta_{\mathrm{ref}}^{\mathrm{CM}}$	1.4 mm iso.	132 × 160 × 112	10:14	9
	CM 5B1P/$\theta_{\mathrm{ref}}^{\mathrm{CM}}$	1.2 mm iso.	156 × 192 × 128	12:38	9
Reduced FoV High Resolution	CM 5B1P/$\theta_{\mathrm{ref}}^{\mathrm{CM}}$	1.2 mm iso.	150 × 192 × 60	5:47	9
	CM 5B1P/$\theta_{\mathrm{ref}}^{\mathrm{CM}}$	1.0 mm iso.	180 × 224 × 72	7:57	9

*Note*: All sequences from Experiment A were acquired with a nominal spatial resolution of 2.0 mm isotropic. The advised parameters are those resulting from running the optimization after fitting the simulation model to the acquired data. A range of $TR_{\text{Burst}}$ is given in square brackets next to the advised “best” such that the simulated SNR efficiency is within 10% of its maximum efficiency. This range is here to mitigate variability effects from inter‐individual microstructure and promote homogeneity of $TR_{\text{Burst}}$ across sequence parametrizations.

#### 2.2.2 General Processing Steps

For each volunteer, we computed an effective $T_{1}$ map ($qT_{1}$) using the MP2RAGE [20] uniform (UNI) image and mono‐component methodology, the $B_{1}^{+}$ field map [21, 27], and in‐house scripts. We generated brain segmentations and parcellations and a brain mask that excludes most of the cerebral‐spinal fluid (CSF) from the denoised and bias field‐corrected UNI image. Moreover, we registered the MNI152 WM probability map and lobe atlas [28] onto each volunteer's UNI image. MT volumes ($MT_{+}$, $MT_{-}$, $MT_{\pm }$, $MT_{\mp }$) were denoised, apodized, zero‐filled, and motion‐corrected as described in Soustelle et al. ([24, 29]). MT ratio [30] (MTR) and ihMT ratio [26] (ihMTR) maps were computed as:

$$M{\mathrm{TR}}_{i}=\frac{MT_{0}-MT_{i}}{MT_{0}},i\in \left\{+,-,\pm ,\mp \right\}$$

$$\begin{array}{ll} \text{ihMTR} & =\frac{\text{ihMT}}{MT_{0}}=\frac{MT_{+}+MT_{-}-MT_{\pm }-MT_{\mp }}{MT_{0}}\\ & \;=M{\mathrm{TR}}_{\pm }+M{\mathrm{TR}}_{\mp }-M{\mathrm{TR}}_{+}-M{\mathrm{TR}}_{-} \end{array}$$

SNR quantification was performed on minimally processed MT volumes (only motion‐correction and, importantly—no denoising). Each UNI, $qT_{1}$, mask, and $B_{1}^{+}$ field maps of a volunteer were registered onto the ihMT images, and each ihMT(R) volume was masked using the product of 4 masks: the volunteer's reference brain mask; a mask of the WM probability atlas for probabilities at or above 95%; the lobe atlas; and a mask of the $B_{1}^{+}$ field map for relative $B_{1}^{+}$ values $B_{1,\mathrm{rel}}^{+}=100\%\pm 2.5\%$ (100% being the nominal transmit field intensity). The resulting ROI averaged about 5000 voxels. ihMT (resp. ihMTR) modes, means, and standard deviations were extracted from fitted Gaussian (resp. skew‐logistic [31]) distributions. The processing steps are further detailed in Supporting Information (Section S3).

#### 2.2.3 Experiment A: SNR‐Efficient Module Optimization and $\text{ihMTR}=f\left(B_{1}^{+}\right)$ Determination

Simulations were iterated twice, initially using literature parameter values and refined by model fitting to acquired experimental data. While absolute signal values differed slightly, trends and optimal experimental parameters were similar after iterating.

##### 2.2.3.1 Optimization of SNR‐Efficient Saturation Modules

SNR‐efficient ihMT sequences were optimized by simulating the expected ihMT signal in WM resulting from the application of MT‐RAGE sequences with different configurations and selecting the ones providing the best ihMT SNR per square root unit of time. Specifications of the resultant sequences are available in Table 1.

MT‐RAGE images were acquired at 2 mm isotropic (iso.) nominal resolution from 4 subjects in 2 sessions. Subjects left the scanner for less than 30 min between sessions. In session 1, SNR‐Efficient ihMT sequences used 4 ALT and 1 CM configurations. In session 2, SNR‐Efficient ihMT sequences used 5 CM and 1 ALT configurations (Table 1). The TR of each sequence was adapted such that the local head SAR would be of about 90% of the first level limitation (local head SAR limit of 20 W/kg over 6 min time‐averaging) for an average in vivo coil load, estimated using a specific anthropomorphic head phantom (SAM, SPEAG, Zurich, Switzerland). The frequency‐alternated and cosine‐modulated configurations common between the sessions (bold in Table 1), hereafter referred to as ${\hat{\theta }}_{\mathrm{ref}}^{\mathrm{ALT}}$ and ${\hat{\theta }}_{\mathrm{ref}}^{\mathrm{CM}}$, were used for test–retest assessment.

**FIGURE 3:**
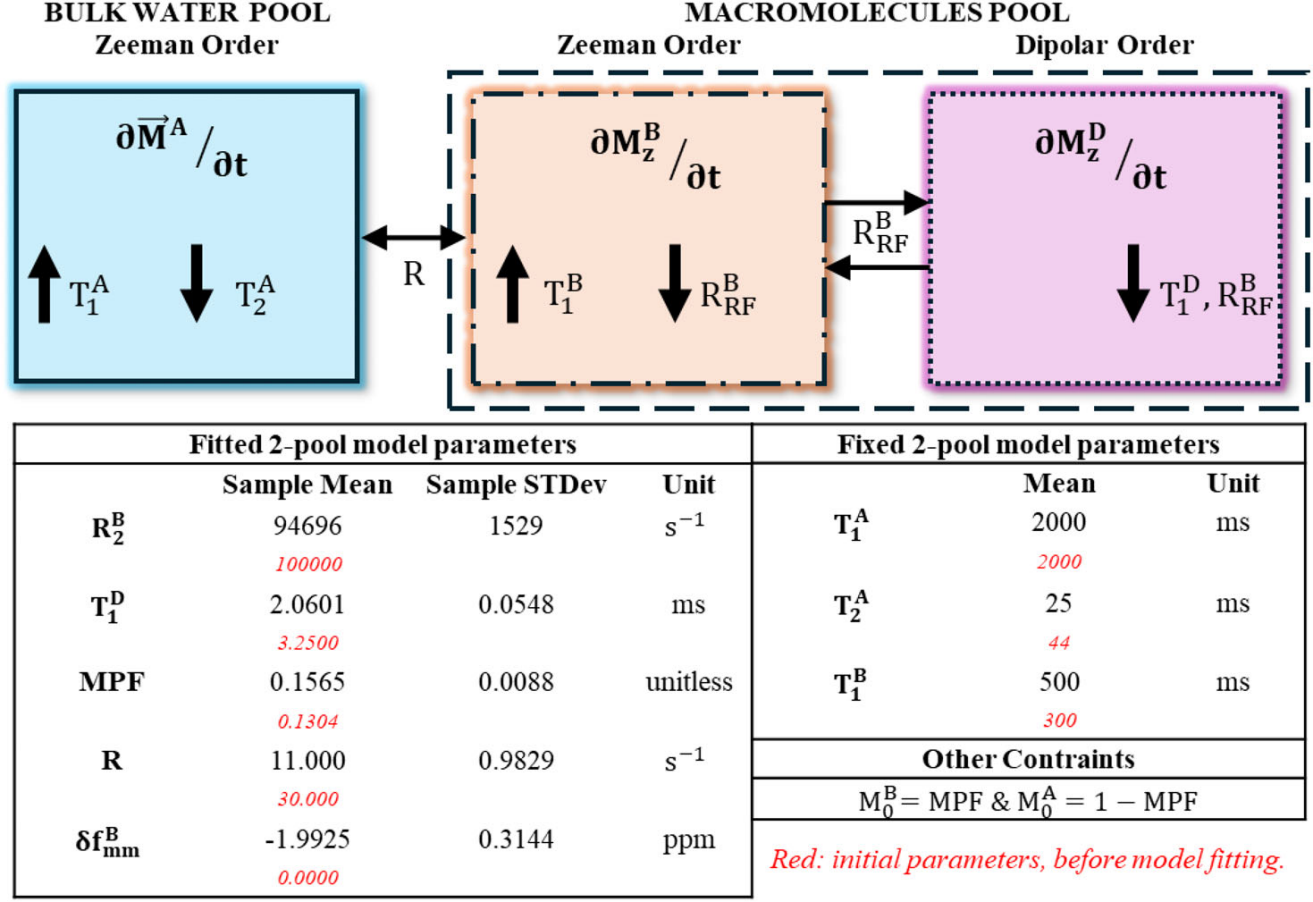
Two‐Pool model of ihMT (5 degrees of freedom) used to fit data and optimize the saturation preparation (Experiment A). The diagram (top) shows the pools and reservoirs that are in exchange and how their concentration is affected by model parameters (arrows). The table (bottom) lists the parameters and other constraints used in the model. Initial values for model parameters (red) were taken from the literature at 3 T to guide the regime of sequences to acquire experimentally based on the results of fitting. Parameters $T_{2}^{A}$ and $T_{1}^{B}$ were not properly constrained by the fit when left free and so were fixed and updated to better reflect estimates at higher field strengths.

Data from Experiment A were segregated into 2 groups: a model fitting group (taken from experiments on N=3 volunteers) and a model validation group (taken from experiments on N=1 volunteer). Note that the duplicate acquisitions between session 1 and 2 used for test–retest assessment were left out to avoid biasing the model toward specific sequences. A biophysical ihMT model, consisting of 2‐pools [3, 18] (bulk water pool and the macromolecular pool made from a Zeeman reservoir coupled to a dipolar reservoir—Figure 3) described by five free biophysical parameters ($T_{2}^{b}$, $T_{1}^{D}$, R, MPF, ${\mathrm{\delta f}}_{\mathrm{mm}}^{b}$, the latter defined as the frequency offset of the macromolecular lineshape with respect to the water lineshape), was fitted to the model fitting dataset's MTR and ihMTR mode values. The modeled system of equations (S1, Section S4) used for fitting was solved numerically using in‐house scripts based on [32].

Confidence intervals were computed using the fitted biophysical parameter means ($\mu_{i}$) and variances ($\sigma_{i}^{2}$). Further details of the fitting process are available in Supporting Information (Section S5).

Validation of the predicted ihMTR signal was performed with $n_{\mathrm{rdm}}=5000$ random parametrization samplings of the model's free parameters generated by assuming each free parameter follows a normal distribution $N\left(\mu_{i},\sigma_{i}\right)$. Each sampled parametrization ${\hat{\theta }}_{k\in \left[1,5000\right]}$ was used to simulate all 9 ihMT sequence configurations (Table 1) to estimate the variability of each sequence in response to a small change of macrostructural properties. The model validation dataset's ihMTR modal values were used for comparison against the predicted ihMTR values. See Supporting Information (Section S6) for details on random samples.

Given the set of sequence parametrizations (Supporting Information, Section S7), the simulated ihMT SNR (where the noise is assumed stationary normally‐distributed with $\mu_{\text{noise}}=0$ and $\sigma_{\text{noise}}=1$) and the associated TR were used to define an optimal RF saturation configuration scheme ${\hat{\theta }}_{\text{optimal saturation}}$ given by:

$${\hat{\theta }}_{\text{optimal saturation}}=\max_{s\in 𝒮}\left(\frac{C\times \mu_{\text{ihMT}}^{s,\text{simulated}}}{\sigma_{\text{noise}}}\frac{1}{\surd \mathrm{TR}}\right)=\max_{s\in 𝒮}\left(\frac{\mu_{\text{ihMT}}^{s,\text{simulated}}}{\surd \mathrm{TR}}\right)$$

With S the set of sequences simulated, $\mu_{\text{ihMT}}^{s,\text{simulated}}$ the ihMT signal predicted by the model for the sequence s, and C a constant that includes, among others, proton density, $T_{2}^{*}$ weighting, and receive coil sensitivity effects, which we set to unity since ${\hat{\theta }}_{\text{optimal saturation}}$ is only ever used intra‐comparatively. Because (thermal) noise distributions were assumed to be the same for all sequences, simulated or acquired, optimizing with respect to SNR efficiency was equivalent to optimizing with respect to the relative SNR efficiency, or more simply, with respect to the relative ihMT efficiency. As such, we used SNR, relative SNR, and ihMT interchangeably in this section.

##### 2.2.3.2 Estimation of ihMTR Dependencies on $B_{1}^{+}$

Using the aforementioned fitted two‐pool model (Figure 3), the ihMT sequence variants were numerically simulated for a range of relative excitation field $B_{1,\mathrm{rel}}^{+}$ values ranging from 5% to 140% its nominal value ($B_{1,\text{nominal}}^{+}$). The resulting data were interpolated using Piecewise Cubic Hermite Interpolation Polynomials (PCHIP) [33] to create $\text{ihMT}R_{s}=f_{s}\left(B_{1}^{+}\right)$ functions tailored specifically to each sequence variant s. Further details on $B_{1}^{+}$ corrections are available in Supporting Information (Section S8).

##### 2.2.3.3 Intra and Inter‐Individual Variabilities Estimations

Two of each SNR‐Efficient ihMT sequences with parametrization ${\hat{\theta }}_{\mathrm{ref}}^{\mathrm{CM}}$ and ${\hat{\theta }}_{\mathrm{ref}}^{\mathrm{ALT}}$ were acquired—one per session, with time interval between both sessions considered sufficiently short to assume that each volunteer presented with the same microstructure and physiological state.

An estimation of the intra‐ and inter‐individual coefficients of variations was obtained by assuming that the mean ihMTR of an individual is sampled from a Gaussian distribution associated with that individual's ROI. Due to the small sample sizes of $n_{\text{intra}}=2$ per sequence per volunteer and $n_{\text{inter}}^{\mathrm{ALT}}=$ $n_{\text{inter}}^{\mathrm{CM}}=4$ volunteers, the correction factor $c_{4}\left(n\right)$ is required to debias the Gaussian‐distributed sample standard deviation estimator [34]. This is further discussed in Supporting Information (Section S9).

#### 2.2.4 Experiment B: Assessment of $B_{1}^{+}$ Inhomogeneity Retrospective Corrections and High Resolution ihMT Imaging

##### 2.2.4.1 $B_{1}^{+}$ Inhomogeneity Retrospective Corrections

During Experiment B, four 2‐mm iso. SNR‐Efficient ihMT sequences were acquired with ${\hat{\theta }}_{\mathrm{ref}}^{\mathrm{CM}}$ on N=1 subject and setting the reference voltage $V_{\mathrm{ref}}$ of the transmission coil to 100%, 80%, 60%, and 40% its nominal value, hereafter referred to as *variable* $V_{\mathrm{ref}}$ sequences. This was to induce a deliberate global $B_{1}^{+}$ scaling in the sample and further assess the $B_{1}^{+}$ correction method. A $B_{1}^{+}$ field map was acquired between acquisition of $V_{\mathrm{ref}}$ 80% and 60% sequences.

Variable $V_{\mathrm{ref}}$ ihMTR volumes were corrected using the processed $B_{1}^{+}$ field map in ihMT space (Section 2.1 for details on the processing of $B_{1}^{+}$ field maps) and f, the $B_{1}^{+}$ dependency of ihMTR, using the following equation:

$$\text{ihMT}R^{\text{corrected}}=\text{ihMT}R^{\text{measured}}\frac{f\left(B_{1,\text{nominal}}^{+}\right)}{f\left(B_{1}^{+,\text{measured}.}\right)}$$

Error from a potentially biased $B_{1}^{+}$ map was estimated over the relative amplitude range $B_{1,\mathrm{rel}}^{+}\in \left[7.5,132.5\right]\%$ assuming a ±5% relative error (e.g., for a nominal $B_{1,\mathrm{rel}}^{+}=60\%$, we assumed a measurement range [57,63]%). Modes and standard deviations of the variable $V_{\mathrm{ref}}$ ihMTR maps before and after retrospective $B_{1}^{+}$ inhomogeneity correction were extracted for comparison purposes.

##### 2.2.4.2 Exploration of High‐Resolution Applications

During Experiment B, additional SNR‐Efficient ihMT sequences using ${\hat{\theta }}_{\mathrm{ref}}^{\mathrm{CM}}$were acquired in a single subject: at 1.2 and 1.4 mm iso. nominal resolutions with a whole brain FOV coverage (full‐FOV), and at 1.0 and 1.2 mm iso. with a thalami‐centered reduced‐FOV coverage. Images from each sequence were independently denoised and motion‐corrected; ihMT(R) volumes were computed and $B_{1}^{+}$‐corrected using the aforementioned process.

##### 2.2.4.3 SNR Estimations

Five MT_0_ sequences, one for each parametrization (full‐FOV: 2, 1.4, 1.2 mm iso.; reduced‐FOV: 1.2, 1.0 mm iso.) were acquired with $V_{\mathrm{ref}}=0\%$, on a single subject. These volumes will be referred to as thermal noise volumes hereafter. The thermal noise volumes were acquired immediately before or after an ihMT sequence sharing the same readout module and were used for noise characterization. This method of estimating noise distributions and SNR quantification comes with a set of hypotheses and statistical subtleties which are further discussed in Supporting Information (Section S10).

The noise distributions were independently fitted as generalized gamma distributions [35, 36]. Modes, means, and standard deviations were extracted from the fits in lieu of using data means and standard deviations.

Computation of SNR was done using the formula [37]

$$\mathrm{SNR}=\frac{\mu_{\text{signal}}}{\sigma_{\text{noise}}}=\frac{\mu_{\text{ihMT}}}{\sqrt{n_{\mathrm{NEX}}^{\text{ihMT}}}\sigma_{\text{noise}}^{\text{thermal}}}=\frac{\mu_{\text{ihMT}}}{2\sigma_{\text{noise}}^{\text{thermal}}}$$

with $n_{\mathrm{NEX}}^{\text{ihMT}}=4$ in our sequence implementation.

## 3 Results

A summary table of ihMTR modes, intra‐individual coefficients of variation (CVs), and estimated SNR for each volunteer in Experiment A and B is given in Supporting Information (Section S11). A representative 3D slice of the MT images for Experiment B's whole‐brain 1.2 mm iso. nominal resolution is available in Supporting Information (Section S12). Due to the point‐spread function (PSF) of the measurement system and image processing (including k‐space trajectory, number of readout segments, partial Fourier saturation, cosine‐apodization), the effective resolution is lower than the nominal resolution. Line profiles of CSF‐masked MT, MTR, and ihMTR images as qualitative proxies for effective contrast resolution are available in Supporting Information (Section S13).

### 3.1 Experiment A: SNR‐Efficient Module Optimization and $\text{ihMTR}=f\left(B_{1}^{+}\right)$ Determination

#### 3.1.1 Optimization of SNR‐Efficient Saturation Modules

**FIGURE 4:**
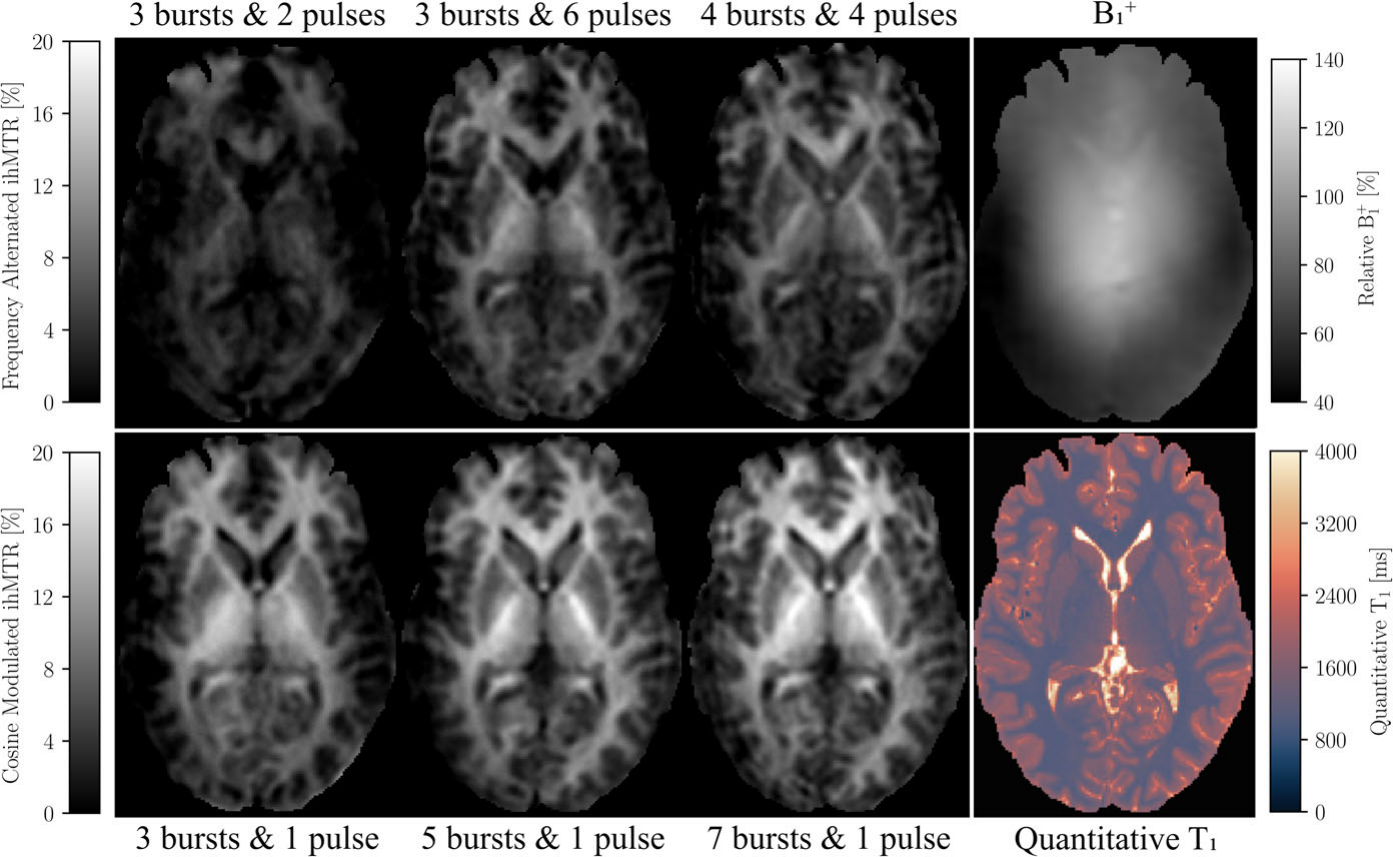
Axial slices of processed ihMTR maps for 3 frequency‐alternated sequences (top left) and 3 cosine‐modulated sequences (bottom left) along with the $B_{1}^{+}$ field map (top right) and the quantitative $T_{1}$ map (bottom right) from a representative volunteer. Every volume was rigid‐registered onto the same visualization space. The $B_{1}^{+}$ field map shows deep field inhomogeneities in peripheral regions of the brain which are visible on the quantitative $T_{1}$ map but characterized by an absence of signal in ihMTR maps. Cosine‐modulated maps all show significantly higher overall signal than frequency‐alternated maps.

Figure 4 presents representative axial non‐$B_{1}^{+}$ corrected ihMTR images derived from sequences of the validation dataset. Signal variations versus number of pulses and number of bursts were observed for both frequency‐alternated and cosine‐modulated ihMTR maps. Cosine‐modulation provides higher ihMTR values than frequency‐alternation. It is clear from these images that peripheral regions of the brain can be deeply affected by $B_{1}^{+}$ inhomogeneity, in particular for ALT sequences (e.g., 3B2P configurations).

**FIGURE 5:**
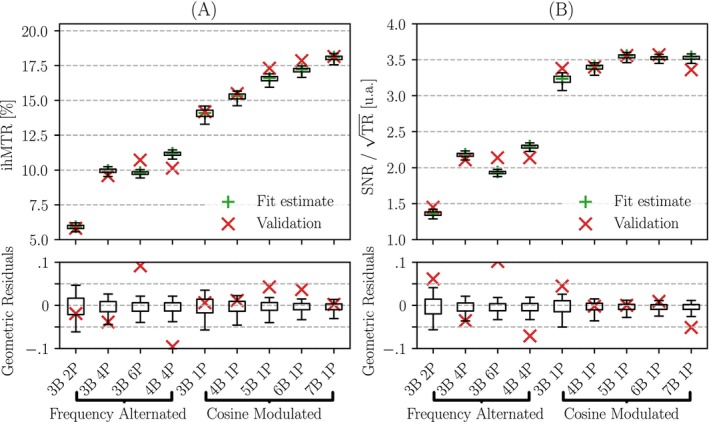
Comparison of ihMTR estimates (top) and its geometric residuals (bottom) for the various sequences based on parameters output from fits to the two‐pool model, which were then applied to the validation dataset. A box plot of the variability in each estimate is overlaid on top. Variability was assessed using $N_{\mathrm{rdm}}=5000$ covariant Gaussian random sets of the free model parameters. Note that the box plot whiskers extend from 2.5% to 97.5% of the full distributions to reject outliers. No $B_{1}^{+}$ correction was applied.

Figure 5A,B shows a box plot of the expected signal by the fitted model and its associated estimated variability compared to the ihMTR signal (resp. SNR efficiency, defined as $\mathrm{SNR}/\sqrt{\mathrm{TR}}$) of the WM sampled from the model validation dataset. We see that the fitted two‐pool model satisfactorily predicts ihMTR values (resp. SNR efficiency) of the model validation dataset with some limitations toward modeling the change in number of bursts in ALT sequences between the 3 bursts and 6 pulses and 4 bursts and 4 pulses (${\hat{\theta }}_{\mathrm{ref}}^{\mathrm{ALT}}$) sequences. The box plots for uncertainty quantifications, extending from 2.5% to 97.5% of the full simulation distributions, show that absolute uncertainties on ihMTR (resp. SNR efficiency) quantification are slightly more pronounced in CM saturation schemes, but show larger ihMTR signal intensity (resp. SNR efficiency). Relative uncertainties show no characteristic differences between CM and ALT saturation schemes. Figure 5B shows that CM sequences offer the best trade‐off between SNR and TA, with the ${\hat{\theta }}_{\mathrm{ref}}^{\mathrm{CM}}$ sequence performing slightly more efficiently than other CM sequences. After model fitting, ihMT sequence simulations (CM: 1–10 bursts and ALT: 3–5 bursts and 2, 4, 6, 8 pulses, each with $TR_{\text{Burst}}\in \left[15,150\right]$ ms) further confirms the ${\hat{\theta }}_{\mathrm{ref}}^{\mathrm{CM}}$ parametrization used in Experiment A as most optimal among those simulated and experimentally feasible (Section S7 of Supporting Information shows the results for all simulated parametrizations regardless of their experimental feasibility, while Figure 5B shows the SNR efficiency for all experimentally feasible parametrizations), indicating that the model parameters found in the literature are consistent up to a scaling in reproducing the trends of ihMT with respect to sequence parametrization. The recommended sequence parametrizations are given in Table 1. Qualitative comparison of images using the acquired and advised sequence parametrizations for whole‐brain CM 5B1P ihMT at 1.4 mm iso. is available in Supporting Information (Section S14).

#### 3.1.2 Estimation of $B_{1}^{+}$ Dependency of ihMTR

**FIGURE 6:**
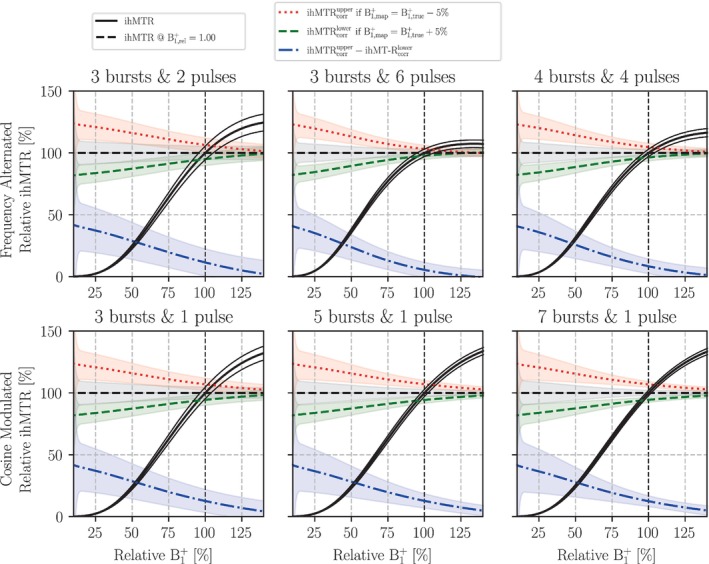
$B_{1}^{+}$ dependency of simulated ihMTR for various sequence configurations. Nominal ihMTR across $B_{1}^{+}$ after perfect correction are shown with dashed black lines. Upper and lower ihMTR post‐$B_{1}^{+}$ correction assuming 5% relative error in $B_{1}^{+}$ are shown in dotted red and dashed green, respectively. The difference between upper and lower ihMTR with 5% relative $B_{1}^{+}$ error is shown in dash‐dotted blue line. Colored bands surrounding each metric (lines) show the ± 95% variability of the metric. *Y*‐axis represents relative ihMTR with respect to the nominal ihMTR at $B_{1,\mathrm{rel}}^{+}=100\%$.

Figure 6 shows the expected relationship between ihMTR and the $B_{1}^{+}$ field intensity for each acquired sequence according to the fitted two‐pool model (thick solid black line). Assuming an arbitrary 5% relative error on the estimation of the $B_{1}^{+}$ field at any given point, the post‐correction ihMTR values (red dotted and green dashed lines) show that corrected ihMTR variability (dash‐dotted blue line) is sufficiently small in $B_{1,\mathrm{rel}}^{+}>80\%$ regions, with less than 25% ihMTR variability. However, corrected ihMTR variability becomes unusably too large as $B_{1}^{+}$ decreases further. Of note, corrections for decreased $B_{1}^{+}$ tend to overshoot the nominal ihMTR. Finally, correction variabilities from model parameter uncertainty grow rapidly as $B_{1}^{+}$ lowers.

Specifically for the ${\hat{\theta }}_{\mathrm{ref}}^{\mathrm{CM}}$ sequence, ihMTR variability after correction (dash‐dotted blue line) at $B_{1,\mathrm{rel}}^{+}=100\%$ is up to $\text{δihMT}R_{\mathrm{rel}}\approx 20\%$ (δihMTR≈3% in absolute ihMTR values) around the nominal ihMTR value (dashed black line) but increases up to $\text{δihMT}R_{\mathrm{rel}}\approx 40\%$ (δihMTR≈6%) for $B_{1,\mathrm{rel}}^{+}=50\%$, that is, a small uncertainty in the $B_{1}^{+}$ value in the low $B_{1}^{+}$ regime propagates to a substantial error in ihMTR. Conversely, the dispersion width tends toward 0 as $B_{1}^{+}$ increases due to the plateauing $\text{ihMTR}=f\left(B_{1}^{+}\right)$.

#### 3.1.3 Intra and Inter‐Individual Variabilities Estimations

The unbiased intra‐individual CVs at 95% Confidence Interval (CI) computed on ihMTR signal measurements vary as $CV_{95\%}^{\mathrm{CM},\text{intra}}\in \left[0.9,4.7\right]\%$ of the signal for CM sequences while they vary as $CV_{95\%}^{\mathrm{ALT},\text{intra}}\in \left[1.9,7.5\right]\%$ of the signal for ALT sequences. Unbiased inter‐individual coefficients of variations were computed to be $CV_{95\%}^{\mathrm{CM},\text{inter}}=3.8\%$ of the signal for CM sequences and $CV_{95\%}^{\mathrm{ALT},\text{inter}}=3.3\%$ of the signal for ALT sequences.

In terms of ihMTR and SNR, these intra‐ and inter‐individual unbiased CVs are of the order of ΔihMTR≈±0.05×ihMTR and ΔSNR≈±0.05×SNR each. In particular, they come to about ΔihMTR≈1% and ΔSNR≈10 per CV for the ${\hat{\theta }}_{\mathrm{ref}}^{\mathrm{CM}}$ configuration.

### 3.2 Experiment B: Assessment of $B_{1}^{+}$ Inhomogeneity Retrospective Corrections and High Resolution ihMT Imaging

#### 3.2.1 $B_{1}^{+}$ Inhomogeneity Retrospective Corrections

**FIGURE 7:**
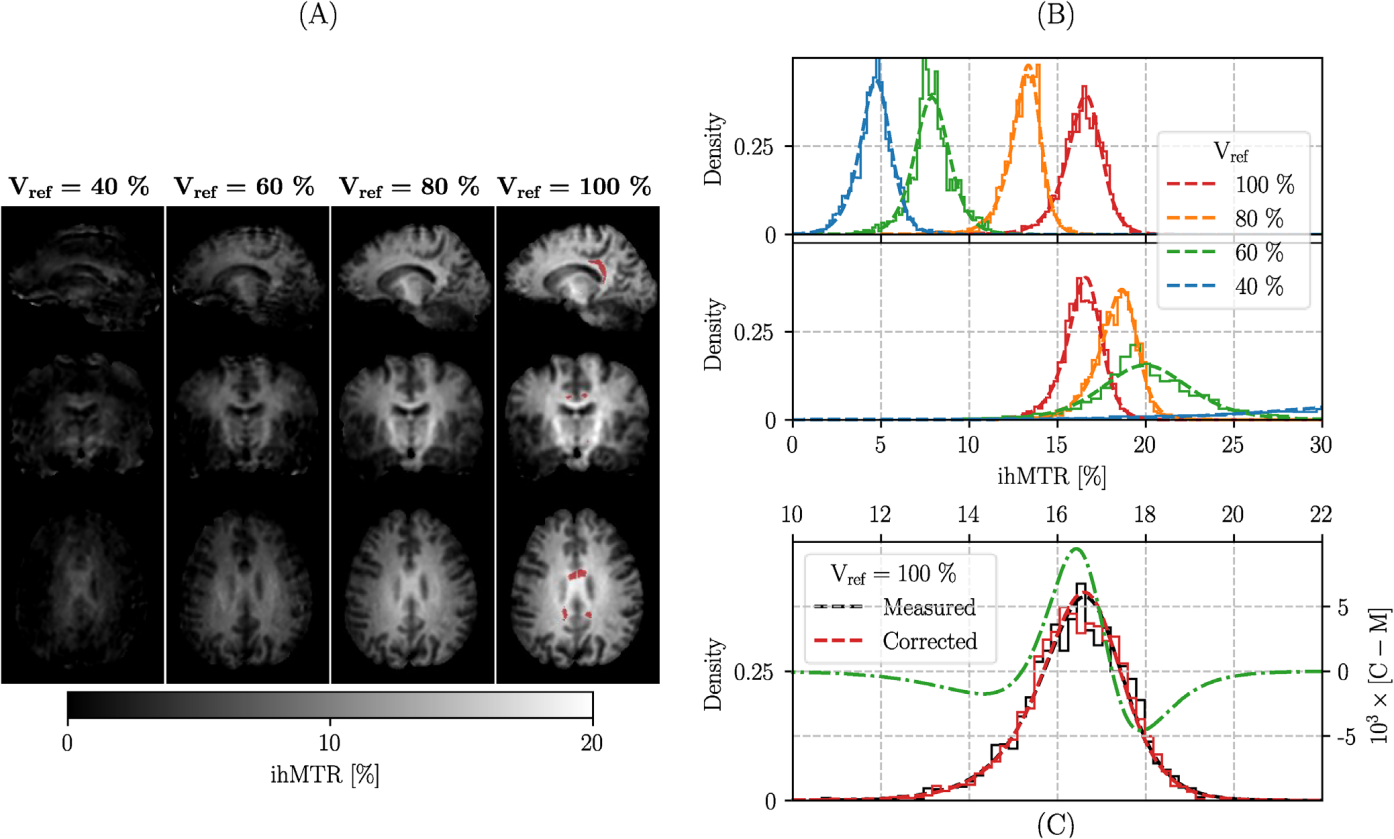
Impact of the $B_{1}^{+}$ correction scheme on ihMTR with the ${\hat{\theta }}_{\mathrm{ref}}^{\mathrm{CM}}$ (CM 5B1P) configuration in a single volunteer. (A) Representative orthogonal ihMTR maps for different nominal $V_{\mathrm{ref}}$ (40% to 100% from left to right) to interrogate $B_{1}^{+}$ inhomogeneities within a single, unchanged, ROI. The ROI based on WM in $V_{\mathrm{ref}}=100\%$ and $B_{1,\mathrm{rel}}^{+}\in \left[97.5,102.5\right]\%$ is overlaid in red. (B) Histograms of ihMTR and fitted skew‐logistic distribution for the single ROI pre (top) and post (bottom) $B_{1}^{+}$ correction. (C) Comparison of the histograms and fitted skew‐logistic distributions pre (dashed black) and post (dashed red) $B_{1}^{+}$ correction for $V_{\mathrm{ref}}=100\%$. The absolute difference between pre (M) and post (C) fitted skew‐logistic distributions is shown with a dash‐dotted green line.

Figure 7A shows representative 3D slices of the ${\hat{\theta }}_{\mathrm{ref}}^{\mathrm{CM}}$ sequence that was acquired at variable nominal $V_{\mathrm{ref}}\in \left\{40,60,80,100\right\}\%$ (left to right). This allowed us to mimic $B_{1}^{+}$ inhomogeneities within a single, unchanged, and microstructurally‐invariant ROI (overlaid in red on the $V_{\mathrm{ref}}=100\%$ image). This ROI was used for distribution analyses of the $B_{1}^{+}$ correction shown in Figure 7B,C.

Figure 7B shows a systematic analysis of the signal distribution pre‐ and post‐correction, revealing a tendency of the model to overshoot the correction factor with decreasing $B_{1}^{+}$, creating highly hyper‐intense regions but few hypo‐intense regions. Conversely, no overshoot was observed for $B_{1,\mathrm{rel}}^{+}=100\%\pm 2.5\%$.

Figure 7C shows that for $B_{1,\mathrm{rel}}^{+}=100\%\pm 2.5\%$, the $B_{1}^{+}$ correction slightly shifts the ihMTR distribution leftward (lower ihMTR).

**FIGURE 8:**
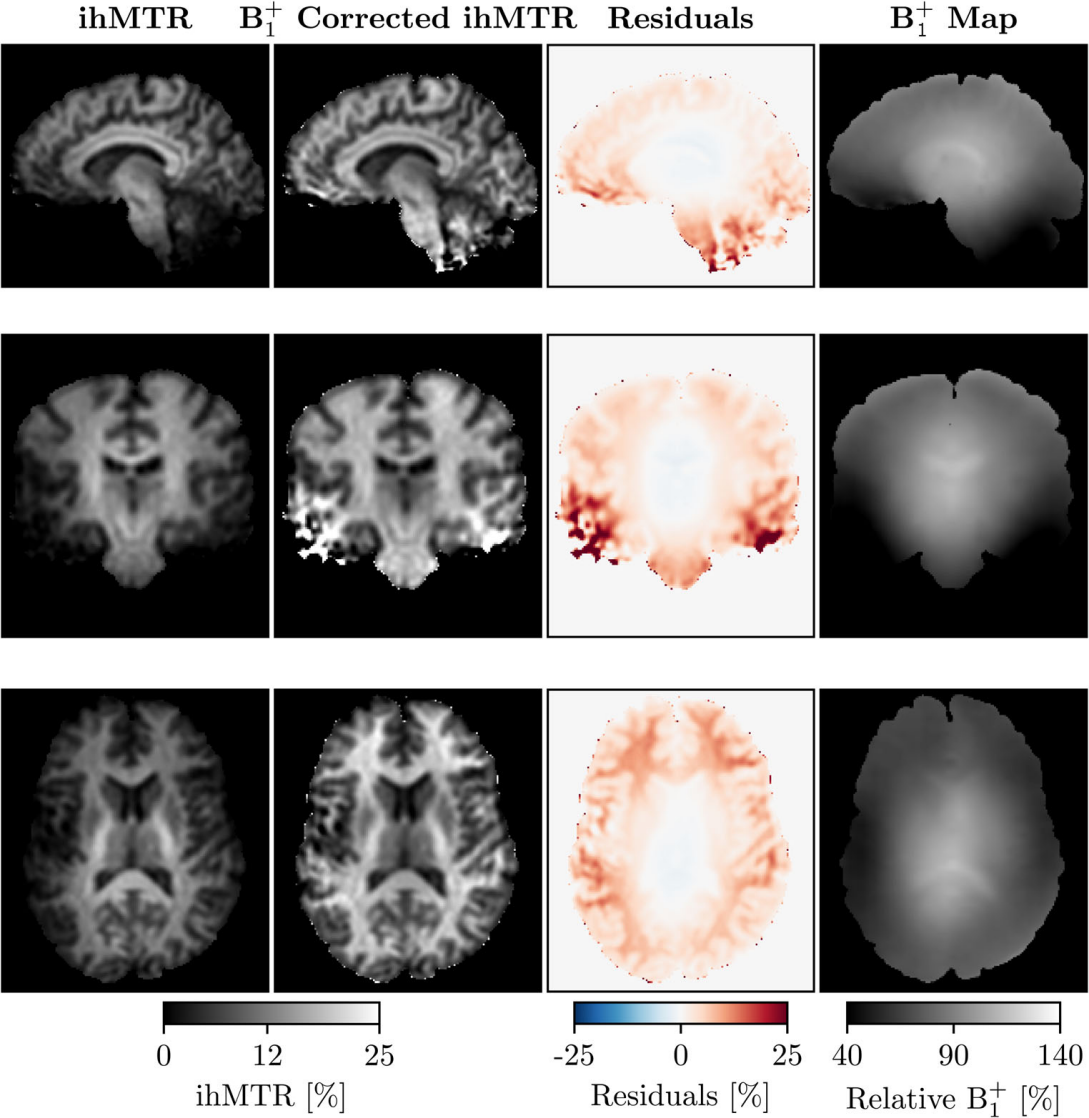
Representative orthogonal CM 5B1P ihMTR maps from the validation dataset without (left column) and with $B_{1}^{+}$ correction (2nd column from left), and the difference between them (2nd column from right) showing the impact of the $B_{1}^{+}$ correction scheme. The associated $B_{1}^{+}$ field map (right column) suggests that this $B_{1}^{+}$ correction scheme is useful in regions with $B_{1,\mathrm{rel}}^{+}\geq 80\%$, but ill‐advised in regions of hypo‐$B_{1}^{+}$ ($B_{1,\mathrm{rel}}^{+}<80\%$).

Figure 8 shows representative 3D ihMTR brain orthogonal slices from the $B_{1}^{+}$ correction validation dataset (Experiment B) before (left) and after (center) the $B_{1}^{+}$ correction algorithm, as well as the associated $B_{1}^{+}$ map (right). The brain regions associated with $B_{1}^{+}$ values above 80% tend to be adequately corrected, however, regions with low $B_{1}^{+}$ (e.g., peripheral regions toward the neck), which show low ihMTR on the pre‐correction image, exhibit strong hyper‐intense signal after correction. The correction fails in the same regions regardless of the optimized ihMT sequence parametrization.

#### 3.2.2 Exploration of High‐Resolution Applications

IhMT sequences with the ${\hat{\theta }}_{\mathrm{ref}}^{\mathrm{CM}}$ configuration, show a signal ihMTR=16% averaged over the WM ROI in the $B_{1,\mathrm{rel}}^{+}=100\%\pm 2.5\%$ range for every resolution and FOV.

**FIGURE 9:**
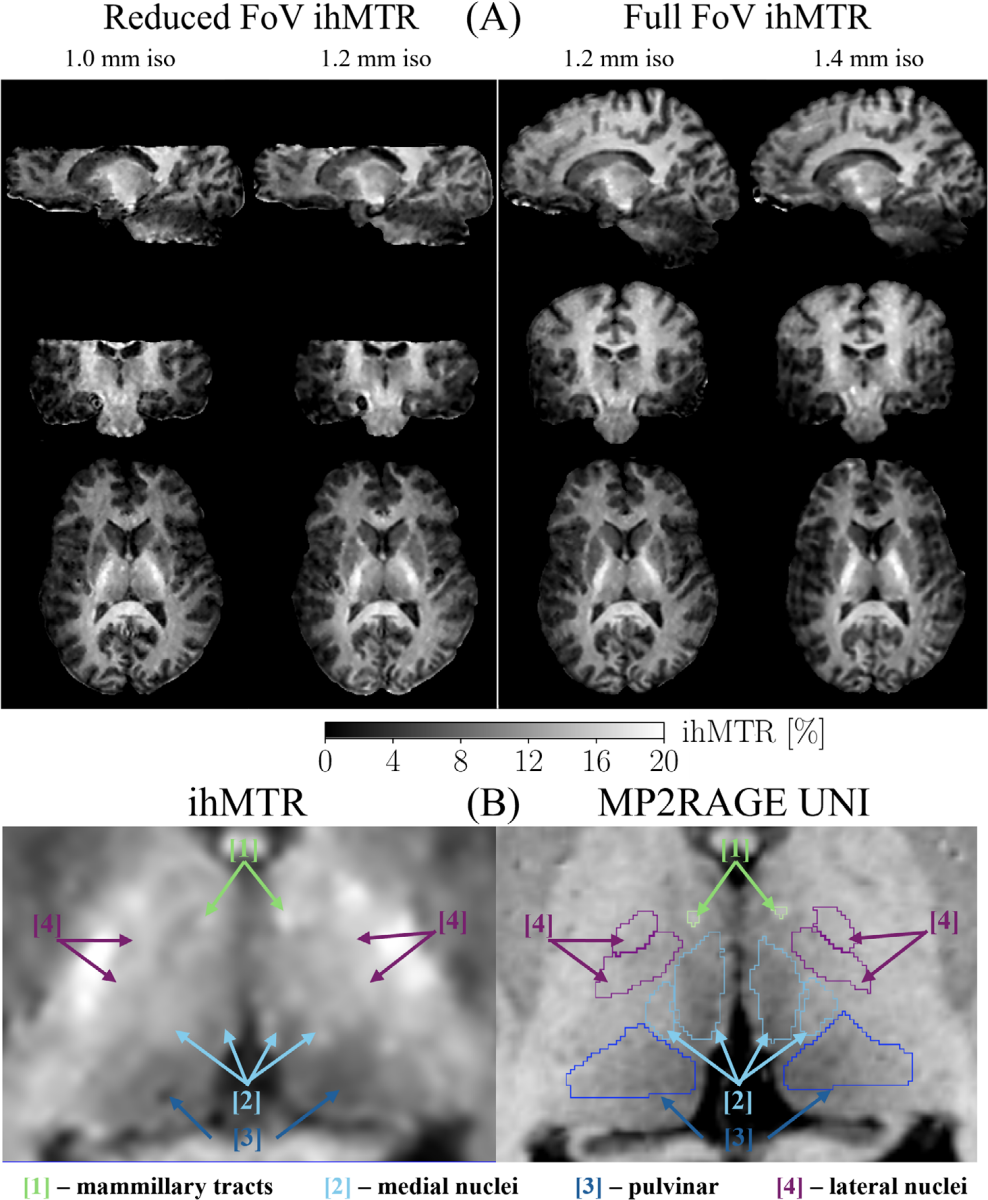
(A) Representative orthogonal high resolution 1 and 1.2 mm iso. reduced‐FOV (left) and 1.2 mm and 1.4 mm iso. full‐FOV (right) ihMTR maps. Images were fully preprocessed (denoising, apodization, zero‐filling, and motion correction) but are shown prior to any $B_{1}^{+}$‐correction scheme. (B) Axial slice comparison of the high resolution 1 mm iso. reduced‐FOV ihMTR map (left) and rigid‐registered MP2RAGE UNI anatomical image (right), overlaid with a contour of the s‐THOMAS atlas. Arrows point to various remarkable thalamic nuclei discussed in the article body. The atlas was not overlaid on the ihMTR map to preserve unbiased contrast visibility.

Figure 9A (top) shows both reduced‐FOV (left panel, 2 columns) and full‐FOV (right panel, 2 columns) ihMTR images. These ihMTR volumes display clear deep gray nuclei, in particular within the thalami. Notably, from Figure 9B (bottom), the pulvinar shows strong contrast relative to adjacent nuclei, consistent with its lower myelination [38]. In contrast, the densely myelinated mammillary tracts in the anterior thalamus are clearly delineated by their high ihMTR values. Subtle contrast is also observable between the medial and lateral thalamic nuclei.

#### 3.2.3 SNR Estimations

Whole‐brain high resolution ${\hat{\theta }}_{\mathrm{ref}}^{\mathrm{CM}}$ ihMT maps show $\mathrm{SNR}={76\;}_{\pm 6\;\left(CV_{95\%}^{\text{inter}}\right)}^{\pm 5\;\left(CV_{95\%}^{\text{intra}}\right)}$ for the 1.4 mm iso. and $\mathrm{SNR}={54\;}_{\pm 4\;\left(CV_{95\%}^{\text{inter}}\right)}^{\pm 3\;\left(CV_{95\%}^{\text{intra}}\right)}$ for the 1.2 mm iso. in $\mathrm{TA}=10^{\prime }14^{\prime \prime }$ and $\mathrm{TA}=12^{\prime }38^{\prime \prime },$ respectively (Figure 9). This is contrasted with $\mathrm{SNR}={135\;}_{\pm 11\;\left(CV_{95\%}^{\text{inter}}\right)}^{\pm 8\;\left(CV_{95\%}^{\text{intra}}\right)}$ and $\mathrm{TA}=5^{\prime }47^{\prime \prime }$ of the equivalent 2 mm isotropic. Reduced‐FOV high resolution ${\hat{\theta }}_{\mathrm{ref}}^{\mathrm{CM}}$ ihMT maps has reduced $\mathrm{SNR}={34\;}_{\pm 3\;\left(CV_{95\%}^{\text{inter}}\right)}^{\pm 2\;\left(CV_{95\%}^{\text{intra}}\right)}$ at 1.2 mm iso., and $\mathrm{SNR}={25\;}_{\pm 2\;\left(CV_{95\%}^{\text{inter}}\right)}^{\pm 2\;\left(CV_{95\%}^{\text{intra}}\right)}$ at 1.0 mm iso. in $\mathrm{TA}=5^{\prime }47^{\prime \prime }$ and $\mathrm{TA}=7^{\prime }57^{\prime \prime },$ respectively. However, the reduced SNR of our reduced‐FOV 1.2 mm (right column of the left panel on Figure 9) compared to our full‐FOV 1.2 mm iso. (left column of the right panel on Figure 9) is not obviously apparent in images and does not prevent high‐resolution reduced‐FOV applications.

## 4 Discussion

### 4.1 Experiment A: SNR‐Efficient Module Optimization and $\text{ihMTR}=f\left(B_{1}^{+}\right)$ Determination

#### 4.1.1 Optimization of SNR‐Efficient Saturation Modules

While the fitted model used to derive the parameters of the optimal saturation module works satisfactorily for most sequences, it shows some medium discrepancies for ALT sequences. However, the validation dataset being N=1 prevents us from ruling out normal statistical variability from the consequences of an overly simplistic modeling of the underlying microstructure [39, 40]. Furthermore, the assumption that the model parameters are sampled from normal distributions in heterogeneous biological tissues can only estimate the order of magnitude of ihMTR variability.

Nevertheless, we still believe results of Figure 5 to be a fair indicator of the reliability of the model, over the investigated range of parameter variations, close to nominal $B_{1,\text{nominal}}^{+}$. The choice of a simple model was motivated by the lack of the much larger dataset necessary to fit parameters in more comprehensive models (e.g., four‐pool model for WM [39, 40]), which goes beyond the scope of this work. We are confident that the ${\hat{\theta }}_{\mathrm{ref}}^{\mathrm{CM}}$ (CM with 5 bursts and 1 pulse per burst) parametrization represents the best trade‐off in terms of SNR/sqrt(TR) for ihMT imaging at 7 T. While ALT is feasible, it suffers from particularly low SNR in hypo‐$B_{1}^{+}$ regions, making this approach hardly viable outside of localized FOV applications.

The significant reduction in scan time from the novel SNR‐Efficient approach to design of the ihMT RF saturation module means an overall improved robustness to motion artifacts, which is crucial to the use of ihMT in patient studies. Moreover, optimizations at ultra‐high fields could be directly applicable to lower field strengths and allow for relaxation of constraints in other places. For example, at $B_{0}=3$ T, it could enable acquisition of whole brain ihMT‐RAGE sequences without using partial Fourier saturation while keeping a low TA˜5 min at 2 mm iso., freeing the choice of encoding trajectory for more stable ones and avoiding partial Fourier artifacts.

#### 4.1.2 Estimation of $B_{1}^{+}$ Dependency of ihMTR

We have extensively characterized the errors propagated by the proposed model‐based $B_{1}^{+}$ correction associated with the sequences in Table 1, and we have shown it works best for regimes of relative $B_{1,\mathrm{rel}}^{+}\geq 80\%$ (Figure 6).

$B_{1}^{+}$ correction error at low relative $B_{1,\mathrm{rel}}^{+}$ could be reduced by using $B_{1}^{+}$ mapping schemes that are more robust below $B_{1,\mathrm{rel}}^{+}=60\%$, where small $\;B_{1}^{+}$ quantification errors propagate non‐linearly to large ihMTR correction errors. Indeed, systematic underestimation of flip angles caused by $T_{1}$ relaxation during gradient‐echo trains in $B_{1}^{+}$ mappings based on the sat‐TFL method is already known [41]. Moreover, fitting the biophysical model generating the corrections on data acquired at various relative $B_{1,\mathrm{rel}}^{+}$ values may capture hypo‐$B_{1}^{+}$ behaviors of ihMTR more faithfully than the model extrapolated from fits to nominal $B_{1}^{+}$ data.

Finally, the variability of the corrections under a change of values in model parameters was studied assuming parameters sampled from a multivariate Gaussian distribution. This may not capture the real variability of some non‐Gaussian parameters (e.g., MPF). Moreover, specific $B_{1}^{+}$ corrections used for quantitative analyses should be derived from models fitted on a ROIs basis (e.g., distinct correction models for white and gray matter), with more generic corrections for qualitative analyses only. A study of specific $B_{1}^{+}$ correction for gray matter (GM) might be performed using the same steps as those presented here for the WM‐optimized correction.

#### 4.1.3 Intra and Inter‐Individual Variabilities Estimations

The unbiased CVs were computed assuming Gaussian‐distributed ihMTR and SNR to simplify the derivation of the small sample bias correction coefficients. Therefore, they are only rough estimates of real intra and inter‐individual variabilities.

Assuming the magnitudes of the sequences' CVs are correct, we can compare them to variations between healthy and pathological tissues found in the literature. For example, Soustelle et al. [7] demonstrated a relative ihMTR decrease of 20% to 40% in active MS lesions compared to normal WM (healthy volunteer) and normal‐appearing WM (MS patient). They also demonstrated a relative ihMTR decrease of about 10% in normal‐appearing WM compared to normal WM. As such, the intra‐individual CV of SNR‐Efficient ihMT sequences being typically less than 5% makes them good candidates to study the evolution of brain tissues in patients. Furthermore, the inter‐individual CV being less than 4% makes them good candidates to study microstructure differences between healthy and pathological cohorts.

### 4.2 Experiment B: Assessment of $B_{1}^{+}$ Inhomogeneity Retrospective Corrections and High Resolution ihMT Imaging

#### 4.2.1 $B_{1}^{+}$ Inhomogeneity Retrospective Corrections

While the retrospective $B_{1}^{+}$ correction proposed here provides some improvement at regimes close to nominal $B_{1,\text{nominal}}^{+}$, it fails at regimes of hypo‐intense $B_{1}^{+}$ common in UHF MRI. Although better estimation of $B_{1}^{+}$ and ihMTR modeling may help improve such corrections, UHF will benefit more from (partial) homogenization of the $B_{1}^{+}$ field during data acquisition, for example, using parallel transmit [42, 43] (pTx) sequences.

Tentative workflow recommendations are as follows: for quantitative analyses, $B_{1}^{+}$ correction should only be used in $B_{1,\mathrm{rel}}^{+}\geq 80\%$ ROIs. For qualitative analyses, $B_{1}^{+}$ correction should only be applied in $B_{1,\mathrm{rel}}^{+}\geq 60\%$, or an explicit warning should indicate $B_{1,\mathrm{rel}}^{+}<60\%$ ROIs are unreliable.

#### 4.2.2 Exploration of High‐Resolution Applications

Of particular interest for research on thalami and their deep gray nuclei, the brain stem, and so on, targeted acquisitions with reduced‐FOV schemes at 1.0–1.2 mm iso. achieve much shorter scan times (TA=5 min 47 s at 1.2 mm iso., TA=7min 57 s at 1.0 mm iso.). IhMTR is well corrected by the retrospective correction in areas where $B_{1}^{+}$ inhomogeneities are less pronounced, which can make these reduced‐FOV acqsuisitions viable for clinical research.

#### 4.2.3 SNR Estimations

With confidence in the fitted model and acknowledging the caveat that extrapolation of the model to parameters beyond those varied under Experiment A may yield inaccurate predictions, we propose the following ihMT saturation module parametrization: 5 bursts ($TR_{\text{burst}}=145$ ms) of 1 cosine‐modulated RF pulse (pw=5 ms, Tukey shaped with cosine fraction $r_{\text{tukey}}=0.2,B_{1}^{+,\text{peak}}=16.3$ μT, $B_{1}^{+,\mathrm{rms}}=15.2$ μT), as the best compromise for SNR efficiency at 7 T.

The SNR of reduced‐FOV acquisitions (SNR=34 at 1.2 mm iso., SNR=25 at 1 mm iso., averaged over the WM ROI) remains sufficient for applications such as deep gray nuclei imaging, which showed fine structures matching MP2RAGE UNI images. Lower SNR compared to their whole‐brain counterpart is expected from the smaller 3D FOV for an equivalent resolution and acceleration scheme [44].

### 4.3 General Considerations

The lower TA of the proposed ihMT sequence allows the acquisition of more ihMT images within a single session than previous state‐of‐the art ihMT sequences. This allows for larger datasets to fit more complex biophysical models [40] at resolutions where these models may be sensitive enough to probe the underlying in vivo microstructure. The lower TA and the applicability of the $B_{1}^{+}$ correction method for a limited $B_{1}^{+}$ inhomogeneity range could make the proposed sequence particularly relevant at lower field strengths (e.g., $B_{0}=3\;T$).

A second avenue for research is the optimization of the signal encoding module to accelerate scans and optimize the PSF [37]. In particular, optimization of the encoding trajectory [45] to be less sensitive to motion [12], and optimization of the partial Fourier saturation scheme for improved PSF.

Finally, the current work was limited to optimizing the MT saturation. It turned out that ihMTR images showed artifacts, most prominently visible in the high‐resolution images of Figure 9. These artifacts can also be seen well on MTR images, available in Supporting Information, Section S12. We hypothesize these artifacts are due to the readout module for two complementary reasons: first, we expect the partial Fourier saturation to add secondary ringing artifacts from the windowing of the MT‐weighted k‐space within the fully acquired k‐space; second, the absence of dummy echoes in the readout echo train which is typical in product centric‐out k‐space trajectories could lead to spurious signals. Future work will focus on a more general readout module optimization.

## 5 Conclusion

We developed a SNR‐Efficient ihMT saturation module for whole brain myelin imaging in clinical research at ultra‐high fields from the principles that stronger pulse amplitudes elicit much stronger ihMT effects, and residual magnetization at the end of a TR will contribute to the overall signal in the following TR—leading to stronger steady‐states reached even at lower saturation duty cycles for sufficiently short TR.

Moreover, we have quantitatively studied variability, impact, and regime of validity of a common model‐based retrospective $B_{1}^{+}$ correction method. We have shown that the $B_{1}^{+}$ correction method significantly increases the variability on the ihMTR signal for $B_{1,\mathrm{rel}}^{+}\leq 80$% the nominal value. We recommend against using this retrospective correction method in strongly hypointense $B_{1}^{+}$ regions.

We have shown feasible the acquisition of ihMT images for resolutions down to 1.2 mm iso. in about 12 min and resolutions down to 2.0 mm iso. in under 6 min for whole brain imaging and down to 1.0 mm iso. in about 6 min for reduced‐FOV imaging with sufficient SNR and low enough CVs to detect pathological ihMTR variations.

Finally, the proposed ihMT saturation module could be trivially translated to lower fields MRI (e.g., $B_{0}=3$ T) for free high returns.

## Funding

This work was supported by A*MIDEX, AMX‐21‐HAN‐01; Agence Nationale de la Recherche, ANR‐22‐CE17‐0060; France Life Imaging, ANR‐11‐INBS‐0006; and France Sclérose en Plaques, FRANCE‐SEP‐24.

## Conflicts of Interest

David C. Alsop is co‐inventor on patents related to ihMT and receives research support from GE Healthcare. Gopal Varma is listed as a co‐inventor on patents related to the ihMT technique. Thomas Troalen is an employee of Siemens Healthineers (Siemens Healthcare SAS, France).

## Supporting information


**Figure S1:** Power deposition as a function of time for the CM 5B1P sequence described in Table 1, centered on a 15 s window (top two) and the full simulation length (bottom two). The top two figures show how the 10 s rolling $B_{1}^{\mathrm{RMS}}$ proxies the rolling 10 s SAR constraints once the MT_0 volume is acquired and the sequence starts depositing RF power as it acquires the MT volumes. The bottom two figures show how the 6 min rolling $B_{1}^{\mathrm{RMS}}$ proxies the rolling 6 min SAR constraints over the full sequence and the impact of Partial Fourier saturation in enabling an SNR‐efficient ihMT acquisition at 7 T. The limited impact of readout excitation pulses on power deposition was neglected for this simulation.
**Figure S2:** (left) correlation matrix of the fitted parameters, (right) sampled model parameters using SciPy's multivariate_normal function and the covariance matrix of the fitted parameters for sample generation and Matplotlib's matshow function and Seaborn's PairGrid function for visualization of the correlation matrix and distribution samplings, respectively. Seaborn's PairGrid function uses the standard kernel density estimation, scatter, and histogram plots with default parameters.
**Figure S3:** Cosine modulated saturation module simulations for the optimization of $\mathrm{SNR}/\sqrt{\mathrm{TR}}$ depending on the number of pulses $N_{P}$ and $TR_{\text{Burst}}$ within a figure and the number of bursts $N_{B}$ across figures.
**Figure S4:** Cosine‐modulated saturation module simulations for the optimization of $\mathrm{SNR}/\sqrt{\mathrm{TR}}$ depending on the number of bursts $N_{B}$ and $TR_{\text{Burst}}$ within a figure and the number of pulses $N_{P}$ across figures.
**Figure S5:** Cosine‐modulated saturation module simulations for the optimization of $\mathrm{SNR}/\sqrt{\mathrm{TR}}$ depending on the number of bursts $N_{B}$ and number of pulses $N_{P}$ given an optimal $TR_{\text{Burst}}$.
**Figure S6:** Frequency‐alternated saturation module simulations for the optimization of $\mathrm{SNR}/\sqrt{\mathrm{TR}}$ depending on the number of pulses $N_{P}$ and $TR_{\text{Burst}}$ within a figure and the number of bursts $N_{B}$ across figures.
**Figure S7:** Frequency‐alternated saturation module simulations for the optimization of $\mathrm{SNR}/\sqrt{\mathrm{TR}}$ depending on the number of bursts $N_{B}$ and $TR_{\text{Burst}}$ within a figure and the number of pulses $N_{P}$ across figures.
**Figure S8:** Frequency‐alternated saturation module simulations for the optimization of $\mathrm{SNR}/\sqrt{\mathrm{TR}}$ depending on the number of bursts $N_{B}$ and number of pulses $N_{P}$ given an optimal $TR_{\text{Burst}}$.
**Figure S9:** Acquired thermal noise map (left) and the associated minimally‐processed ihMT map (right) for the ihMTR CM5B1P at 2.0 mm iso.
**Figure S10:** MT images of whole‐brain CM 5B1P at 1.2 mm iso. in arbitrary DICOM scale, all within the same range. No $B_{1}^{+}$ correction was applied.
**Figure S11:** MTR images of whole‐brain CM 5B1P at 1.2 mm iso. (MT_0) in arbitrary DICOM scale, (MTR) scaled from [0, 60]%. No $B_{1}^{+}$ correction was applied.
**Figure S12:** Line profiles of CSF‐masked MT images for the CM 5B1P 1.2 mm iso. whole‐brain sequence. From left to right, contrasts are $MT_{0}$, $MT_{s}^{+}$, $MT_{s}^{-}$, $MT_{d}^{\pm }$, $MT_{d}^{\mp }$. Visually, the images shown in the top row may be stretched vertically and horizontally, fitting the graph frames to make comparison between columns more straightforward.
**Figure S13:** Line profiles of CSF‐masked MTR images for the CM 5B1P 1.2 mm iso. whole‐brain sequence. From left to right, contrasts are $MT_{0}$, $M{\mathrm{TR}}_{s}^{+}$, $M{\mathrm{TR}}_{s}^{-}$, $M{\mathrm{TR}}_{d}^{\pm }$, $M{\mathrm{TR}}_{d}^{\mp }$. Visually, the images shown in the top row may be stretched vertically and horizontally, fitting the graph frames to make comparison between columns more straightforward.
**Figure S14:** Line profiles of CSF‐masked ihMTR images. Whole‐brain are the 3 left‐most columns. Reduced FOV are the 2 right‐most columns. Images are not registered onto each other so that no blurring from registration occurs. Visually, the images shown in the top row may be stretched vertically and horizontally, fitting the graph frames to make comparison between columns more straightforward.
**Figure S15:** Representative axial slice of ihMTR images (MT preparation: cosine‐modulated 5 bursts 1 pulse) with previously acquired parametrization (left) and advised parametrization (right) showing limited signal intensity differences.
**Table S1:** Software versions.
**Table S2:** Quantitative metrics (ihMTR and SNR) for Experiment A.
**Table S3:** Quantitative metrics (ihMTR and SNR) for Experiment B.

## Data Availability

The data that support the findings of this study are available from the corresponding author upon reasonable request.

## References

[1] A. P. Manning , K. L. Chang , A. L. MacKay , and C. A. Michal , “The Physical Mechanism of “Inhomogeneous” Magnetization Transfer MRI,” Journal of Magnetic Resonance 274 (2017): 125–136, 10.1016/j.jmr.2016.11.013. 27918896

[2] G. Varma , G. Duhamel , C. De Bazelaire , and D. C. Alsop , “Magnetization Transfer From Inhomogeneously Broadened Lines: A Potential Marker for Myelin,” Magnetic Resonance in Medicine 73, no. 2 (2015): 614–622, 10.1002/mrm.25174. 24604578 PMC4378005

[3] G. Varma , O. M. Girard , V. H. Prevost , A. K. Grant , G. Duhamel , and D. C. Alsop , “Interpretation of Magnetization Transfer From Inhomogeneously Broadened Lines (ihMT) in Tissues as a Dipolar Order Effect Within Motion Restricted Molecules,” Journal of Magnetic Resonance 260 (2015): 67–76, 10.1016/j.jmr.2015.08.024. 26408956

[4] G. Duhamel , V. H. Prevost , M. Cayre , et al., “Validating the Sensitivity of Inhomogeneous Magnetization Transfer (ihMT) MRI to Myelin With Fluorescence Microscopy,” NeuroImage 199 (2019): 289–303, 10.1016/j.neuroimage.2019.05.061. 31141736

[5] H. Rasoanandrianina , A. M. Grapperon , M. Taso , et al., “Region‐Specific Impairment of the Cervical Spinal Cord (SC) in Amyotrophic Lateral Sclerosis: A Preliminary Study Using SC Templates and Quantitative MRI (Diffusion Tensor Imaging/Inhomogeneous Magnetization Transfer),” NMR in Biomedicine 30, no. 12 (2017): e3801, 10.1002/nbm.3801. 28926131

[6] E. Van Obberghen , S. Mchinda , A. le Troter , et al., “Evaluation of the Sensitivity of Inhomogeneous Magnetization Transfer (ihMT) MRI for Multiple Sclerosis,” AJNR. American Journal of Neuroradiology 39, no. 4 (2018): 634–641, 10.3174/ajnr.A5563. 29472299 PMC7410781

[7] L. Soustelle , S. Mchinda , A. Hertanu , et al., “Inhomogeneous Magnetization Transfer (ihMT) Imaging Reveals Variable Recovery Profiles of Active MS Lesions According to Size and Localization,” Imaging Neuroscience 2 (2024): imag–2–00235, 10.1162/imag_a_00235. PMC12272197 40800347

[8] M. E. Ladd , P. Bachert , M. Meyerspeer , et al., “Pros and Cons of Ultra‐High‐Field MRI/MRS for Human Application,” Progress in Nuclear Magnetic Resonance Spectroscopy 109 (2018): 1–50, 10.1016/j.pnmrs.2018.06.001. 30527132

[9] O. M. Girard , V. H. Prevost , G. Varma , P. J. Cozzone , D. C. Alsop , and G. Duhamel , “Magnetization Transfer From Inhomogeneously Broadened Lines (ihMT): Experimental Optimization of Saturation Parameters for Human Brain Imaging at 1.5 Tesla: Optimizing Saturation Parameters for ihMT Brain Imaging at 1.5T,” Magnetic Resonance in Medicine 73, no. 6 (2015): 2111–2121, 10.1002/mrm.25330. 24962257

[10] L. P. Panych and B. Madore , “The Physics of MRI Safety,” Magnetic Resonance Imaging 47, no. 1 (2018): 28–43, 10.1002/jmri.25761. 28543948

[11] T. M. Fiedler , M. E. Ladd , and S. Orzada , “Local and Whole‐Body SAR in UHF Body Imaging: Implications for SAR Matrix Compression,” Magnetic Resonance in Medicine 93, no. 2 (2025): 842–849, 10.1002/mrm.30306. 39301784 PMC11604848

[12] M. Bekiesińska‐Figatowska , “Artifacts in Magnetic Resonance Imaging,” Polish Journal of Radiology 80 (2015): 93–106, 10.12659/PJR.892628. 25745524 PMC4340093

[13] M. Park , H. Noh , and N. Park , “Mitigation of B1+ Inhomogeneity for Ultra‐High‐Field Magnetic Resonance Imaging: Hybrid Mode Shaping With Auxiliary EM Potential,” Scientific Reports 10, no. 1 (2020): 11752, 10.1038/s41598-020-68651-6. 32678182 PMC7366730

[14] R. Pohmann , O. Speck , and K. Scheffler , “Signal‐To‐Noise Ratio and MR Tissue Parameters in Human Brain Imaging at 3, 7, and 9.4 Tesla Using Current Receive Coil Arrays,” Magnetic Resonance in Medicine 75, no. 2 (2016): 801–809, 10.1002/mrm.25677. 25820458

[15] C. D. Rowley , J. S. W. Campbell , Z. Wu , et al., “A Model‐Based Framework for Correcting Inhomogeneity Effects in Magnetization Transfer Saturation and Inhomogeneous Magnetization Transfer Saturation Maps,” Magnetic Resonance in Medicine 86, no. 4 (2021): 2192–2207, 10.1002/mrm.28831. 33956348

[16] F. Munsch , G. Varma , M. Taso , et al., “Characterization of the Cortical Myeloarchitecture With Inhomogeneous Magnetization Transfer Imaging (ihMT),” NeuroImage 225 (2021): 117442, 10.1016/j.neuroimage.2020.117442. 33039620

[17] M. Taso , F. Munsch , O. M. Girard , G. Duhamel , D. C. Alsop , and G. Varma , “ fast‐spin‐echo Versus Rapid gradient‐echo for 3d magnetization‐prepared Acquisitions: Application to Inhomogeneous Magnetization Transfer,” Magnetic Resonance in Medicine 89 (2023): 550–564, 10.1002/mrm.29461. 36306334 PMC10848167

[18] G. Varma , F. Munsch , B. Burns , et al., “Three‐Dimensional Inhomogeneous Magnetization Transfer With Rapid Gradient‐Echo (3D ihMTRAGE) Imaging,” Magnetic Resonance in Medicine 84, no. 6 (2020): 2964–2980, 10.1002/mrm.28324. 32602958 PMC7722070

[19] C. D. Rowley , J. S. W. Campbell , I. R. Leppert , M. C. Nelson , G. B. Pike , and C. L. Tardif , “Optimization of Acquisition Parameters for Cortical Inhomogeneous Magnetization Transfer (ihMT) Imaging Using a Rapid Gradient Echo Readout,” Magnetic Resonance in Medicine 90, no. 5 (2023): 1762–1775, 10.1002/mrm.29754. 37332194

[20] J. P. Marques , T. Kober , G. Krueger , W. van der Zwaag , P. F. Van de Moortele , and R. Gruetter , “MP2RAGE, a Self Bias‐Field Corrected Sequence for Improved Segmentation and T1‐Mapping at High Field,” NeuroImage 49, no. 2 (2010): 1271–1281, 10.1016/j.neuroimage.2009.10.002. 19819338

[21] A. Massire , C. Seiler , T. Troalen , et al., “T1‐Based Synthetic Magnetic Resonance Contrasts Improve Multiple Sclerosis and Focal Epilepsy Imaging at 7 T,” Investigative Radiology 56, no. 2 (2021): 127–133, 10.1097/RLI.0000000000000718. 32852445

[22] S. Chung , D. Kim , E. Breton , and L. Axel , “Rapid *B* _1_ ^+^ Mapping Using a Preconditioning RF Pulse With TurboFLASH Readout,” Magnetic Resonance in Medicine 64, no. 2 (2010): 439–446, 10.1002/mrm.22423. 20665788 PMC2929762

[23] S. Mchinda , G. Varma , V. H. Prevost , et al., “Whole Brain Inhomogeneous Magnetization Transfer (ihMT) Imaging: Sensitivity Enhancement Within a Steady‐State Gradient Echo Sequence,” Magnetic Resonance in Medicine 79, no. 5 (2018): 2607, 10.1002/mrm.26907. 28940355

[24] L. Soustelle , T. Troalen , A. Hertanu , et al., “A Strategy to Reduce the Sensitivity of Inhomogeneous Magnetization Transfer (ihMT) Imaging to Radiofrequency Transmit Field Variations at 3 T,” Magnetic Resonance in Medicine 87, no. 3 (2022): 1346–1359, 10.1002/mrm.29055. 34779020

[25] F. J. Harris , “On the Use of Windows for Harmonic Analysis With the Discrete Fourier Transform,” Proceedings of the IEEE 66, no. 1 (1978): 51–83, 10.1109/PROC.1978.10837.

[26] D. C. Alsop , E. Ercan , O. M. Girard , et al., “Inhomogeneous Magnetization Transfer Imaging: Concepts and Directions for Further Development,” NMR in Biomedicine 36, no. 6 (2022): e4808, 10.1002/nbm.4808. 35916067

[27] J. P. Marques and R. Gruetter , “New Developments and Applications of the MP2RAGE Sequence ‐ Focusing the Contrast and High Spatial Resolution R1 Mapping,” PLoS One 8, no. 7 (2013): e69294, 10.1371/journal.pone.0069294. 23874936 PMC3712929

[28] D. L. Collins , A. P. Zijdenbos , W. F. C. Baaré , and A. C. Evans , “ANIMAL+INSECT: Improved Cortical Structure Segmentation,” in Information Processing in Medical Imaging, vol. 1613, ed. A. Kuba , M. Šáamal , and A. Todd‐Pokropek (Springer Berlin Heidelberg, 1999), 210–223, 10.1007/3-540-48714-X_16.

[29] L. Soustelle , J. Lamy , A. Le Troter , et al., “A Motion Correction Strategy for Multi‐Contrast Based 3D Parametric Imaging: Application to Inhomogeneous Magnetization Transfer (ihMT),” bioRxiv (2020): 2020.09.11.292649, 10.1101/2020.09.11.292649.

[30] R. M. Henkelman , G. J. Stanisz , and S. J. Graham , “Magnetization Transfer in MRI: A Review,” NMR in Biomedicine 14, no. 2 (2001): 57–64, 10.1002/nbm.683. 11320533

[31] N. L. Johnson , S. Kotz , and N. Balakrishnan , Continuous Univariate Distributions, 2nd ed. (Wiley, 1995).

[32] D. K. Müller , A. Pampel , and H. E. Möller , “Matrix‐Algebra‐Based Calculations of the Time Evolution of the Binary Spin‐Bath Model for Magnetization Transfer,” Journal of Magnetic Resonance 230 (2013): 88–97, 10.1016/j.jmr.2013.01.013. 23454578

[33] F. N. Fritsch and J. Butland , “A Method for Constructing Local Monotone Piecewise Cubic Interpolants,” SIAM Journal on Scientific and Statistical Computing 5, no. 2 (1984): 300–304, 10.1137/0905021.

[34] W. H. Holtzman , “The Unbiased Estimate of the Population Variance and Standard Deviation,” American Journal of Psychology 63, no. 4 (1950): 615, 10.2307/1418879. 14790030

[35] E. W. Stacy , “A Generalization of the Gamma Distribution,” Annals of Mathematical Statistics 33, no. 3 (1962): 1187–1192, 10.1214/aoms/1177704481.

[36] S. Aja‐Fernández , A. Tristán‐Vega , and W. S. Hoge , “Statistical Noise Analysis in GRAPPA Using a Parametrized Noncentral Chi Approximation Model,” Magnetic Resonance in Medicine 65, no. 4 (2011): 1195–1206, 10.1002/mrm.22701. 21413083 PMC3955201

[37] J. Bushberg , J. A. Seibert , E. M. Leidholdt , and J. M. Boone , The Essential Physics of Medical Imaging, 4th ed. (Wolters Kluwer Medical, 2021).

[38] K. Min , B. Sohn , W. J. Kim , et al., “A Human Brain Atlas of *χ*‐Separation for Normative Iron and Myelin Distributions,” NMR in Biomedicine 37, no. 12 (2024): e5226, 10.1002/nbm.5226. 39162295

[39] M. H. Lam , M. Novoselova , A. Yung , et al., “Interpretation of Inhomogeneous Magnetization Transfer in Myelin Water Using a Four‐Pool Model With Dipolar Reservoirs,” Magnetic Resonance in Medicine 94, no. 1 (2025): 278–292, 10.1002/mrm.30465. 39963772 PMC12021340

[40] N. Wallstein , A. Pampel , R. Müller , C. Jäger , M. Morawski , and H. E. Möller , “An Unconstrained Four Pool Model Analysis of Proton Relaxation and Magnetization Transfer in Ex Vivo White Matter,” Scientific Reports 15, no. 1 (2025): 4354, 10.1038/s41598-025-87362-4. 39910188 PMC11799436

[41] R. Pohmann and K. Scheffler , “A Theoretical and Experimental Comparison of Different Techniques for *B* _1_ Mapping at Very High Fields,” NMR in Biomedicine 26, no. 3 (2013): 265–275, 10.1002/nbm.2844. 22972684

[42] C. M. Deniz , “Parallel Transmission for Ultrahigh Field MRI,” Topics in Magnetic Resonance Imaging 28, no. 3 (2019): 159–171, 10.1097/RMR.0000000000000204. 31188274 PMC7039313

[43] D. Leitão , R. Tomi‐Tricot , P. Bridgen , et al., “Parallel Transmit Pulse Design for Saturation Homogeneity (PUSH) for Magnetization Transfer Imaging at 7t ,” Magnetic Resonance in Medicine 88, no. 1 (2022): 180–194, 10.1002/mrm.29199. 35266204 PMC9315051

[44] R. W. Brown , Y. C. N. Cheng , E. M. Haacke , M. R. Thompson , and R. Venkatesan , Magnetic Resonance Imaging: Physical Principles and Sequence Design, Second ed. (John Wiley & Sons, Inc, 2014).

[45] R. Mezrich , “A Perspective on K‐Space,” Radiology 195, no. 2 (1995): 297–315, 10.1148/radiology.195.2.7724743. 7724743

