# Supplementary material for: SNR‐Efficient Inhomogeneous Magnetization Transfer (ihMT) for Clinical Applications at 7 T
Source: Magn Reson Med. 2026 May 11;96(3):1162–77. doi: 10.1002/mrm.70419 (PMC13327452; doi:10.1002/mrm.70419)
Supplement: Supplementary file 1 — Figure S1: Power deposition as a function of time for the CM 5B1P sequence described in Table 1, centered on a 15 s window (top two) and the full simulation length (bottom two). The top two figures show how the 10 s rolling B1RMS proxies the rolling 10 s SAR constraints once the MT_0 volume is acquired and the sequence starts depositing RF power as it acquires the MT volumes. The bottom two figures show how the 6 min rolling B1RMS proxies the rolling 6 min SAR constraints over the full sequence and the impact of Partial Fourier saturation in enabling an SNR‐efficient ihMT acquisition at 7 T. The limited impact of readout excitation pulses on power deposition was neglected for this simulation. Figure S2: (left) correlation matrix of the fitted parameters, (right) sampled model parameters using SciPy's multivariate_normal function and the covariance matrix of the fitted parameters for sample generation and Matplotlib's matshow function and Seaborn's PairGrid function for visualization of the correlation matrix and distribution samplings, respectively. Seaborn's PairGrid function uses the standard kernel density estimation, scatter, and histogram plots with default parameters. Figure S3: Cosine modulated saturation module simulations for the optimization of SNR/TR depending on the number of pulses NP and TRBurst within a figure and the number of bursts NB across figures. Figure S4: Cosine‐modulated saturation module simulations for the optimization of SNR/TR depending on the number of bursts NB and TRBurst within a figure and the number of pulses NP across figures. Figure S5: Cosine‐modulated saturation module simulations for the optimization of SNR/TR depending on the number of bursts NB and number of pulses NP given an optimal TRBurst. Figure S6: Frequency‐alternated saturation module simulations for the optimization of SNR/TR depending on the number of pulses NP and TRBurst within a figure and the number of bursts NB across figures. Figure S7: Frequency‐alternated s [file MRM-96-1162-s001.docx]

# Supplementary Materials

## Software versions

| Framework | Software | Version | References |
| --- | --- | --- | --- |
| Anaconda environment | Conda | 23.3.1 | ^62,63^ |
|  | Python | 3.12.3 | ^64^ |
|  | NumPy | 2.2.4 | ^82^ |
|  | SciPy | 1.15.3 | ^69^ |
|  | Matplotlib | 3.10.5 | ^71,72^ |
|  | Seaborn | 0.13.2 | ^83^ |
|  | MRtrix3 | 3.0.5 | ^65–68^ |
| ihMT Proc | | Commit #0359430 | github.com/lsoustelle/ihmt_proc/tree/0359430 |
| MATLAB | | 24.2.0.2773142 | ^77^ |
| C++ | | 17 | ^46^ |
| g++ | | Ubuntu 13.1.0-8ubuntu1~20.04.2 | gcc.gnu.org |
| Eigen | | 3.3.7 | eigen.tuxfamily.org |
| Boost | | 1.82.0 | ^47^ |
| libInterpolate | | 2.6.4 | github.com/CD3/libInterpolate/tree/2.6.4 |
| JSON for Modern C++ | | 3.11.3 | github.com/nlohmann/json/tree/v3.11.3 |
| RNifti | | 1.6.0 | github.com/jonclayden/RNifti/tree/v1.6.0 |
| Insight Segmentation and Registration Toolkit (ITK) | | 5.3.0 | ^50,51^ |
| Advanced Normalization Tools (ANTs) | | v2.4.2.post24-gb772b65 | ^48,49,52,53,54(p14)^ |
| FMRIB Software Library (FSL) | | 6.0.5:9e026117 | ^57^ |
| FreeSurfer | | freesurfer-linux-ubuntu20_x86_64-7.3.2-20220804-6354275 | ^55,56,58,59^ |
| ITK-Snap | | 4.2.2 | ^70^ |

Table S1. Software versions.

## $\hat{\theta}_{\mathrm{ref}}^{\mathrm{CM}}$ (CM 5B1P) sequence chronogram


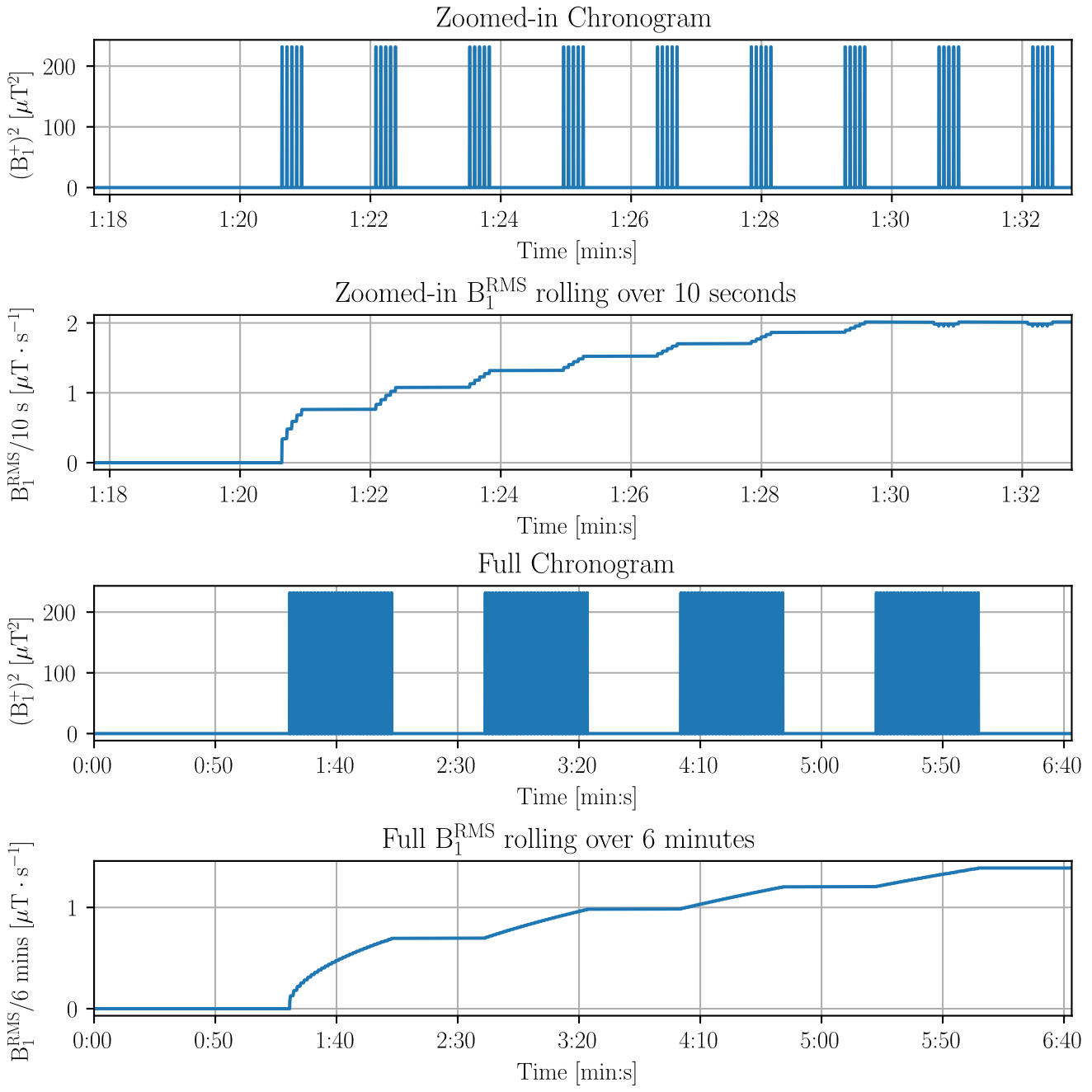


Figure S1. Power deposition as a function of time for the CM 5B1P sequence described in Table 1, centered on a 15 s window (top two) and the full simulation length (bottom two). The top two figures show how the 10 s rolling $B_{1}^{RMS}$ proxies the rolling 10 s SAR constraints once the MT_0 volume is acquired and the sequence starts depositing RF power as it acquires the MT volumes. The bottom two figures show how the 6 mins rolling $B_{1}^{RMS}$ proxies the rolling 6 mins SAR constraints over the full sequence and the impact of Partial Fourier saturation in enabling an SNR-efficient ihMT acquisition at 7 T. The limited impact of readout excitation pulses on power deposition was neglected for this simulation.

The zoomed-in chronogram and 10 s rolling $B_{1}^{\mathrm{RMS}}$ (top two rows) show the effect of the single CM pulse across the 5 bursts during each TR of the $\hat{\theta}_{\mathrm{ref}}^{\mathrm{CM}}$ sequence when the sequence starts to acquire the first MT volume. Notably, we see a transient rise in $B_{1}^{\mathrm{RMS}}$ until 10 s passed after the first pulse, after which $B_{1}^{\mathrm{RMS}}$ reaches a periodic state below the 10 s $B_{1}^{\mathrm{RMS}}$ limit fixed by SAR regulations.

The full chronogram and 6 mins rolling $B_{1}^{\mathrm{RMS}}$ (bottom two rows) show the ordering of the acquired volumes in the ihMT sequence (a single $MT_{0}$ followed by 4 MT volumes), as well as the impact of the partial Fourier saturation scheme on sequence (lack of MT pulses for half the TA of each MT volume, e.g., between 3:25 and 4:00). Notably, we see a transient rise in $B_{1}^{\mathrm{RMS}}$ when the sequence starts acquiring MT volumes, until partial Fourier saturation turns on where $B_{1}^{\mathrm{RMS}}$ stays steady until the next MT volume starts being acquired. This is repeated until the end of the sequence as the ihMT sequence does not last long enough to reach a full 6 mins between the first and the last MT pulse.

## Image processing

All software specifications are listed in Table S1. Specifically, all Python software libraries were installed in a single conda environment. Thereafter, a detailed account of the processing steps and tools used throughout this study.

MP2RAGE uniform (UNI) images were used to compute quantitative T_1_ (qT_1_) maps^20^ using C++^46^ in-house scripts based on the Eigen, Boost^47^, Advanced Normalization Tools (ANTs)^48,49^, Insight Segmentation and Registration Toolkit^50,51^ (ITK), and RNifti libraries, relying on a bijective 2D lookup-table (UNI, T_1_) calculated for the prescribed protocol and considering a single water pool signal model, as done in Massire A. (2021)^21^, and assuming a homogenous $B_{1}^{+}$ field.

For each volunteer, the unsaturated image from the $B_{1}^{+}$ field mapping sequence was rigid-registered onto the qT_1_ map using ANTs *antsRegistration*^52–54^. The $B_{1}^{+}$ field map was then registered onto the qT_1_ map using the transformation matrix from the previous registration and ANTs’ *antsApplyTransforms*. Registered $B_{1}^{+}$ field maps were further smoothed using ANTs’ *SmoothImage* with a $3\times1\times1$ Gaussian kernel and were used along with the original UNI maps to generate the following 3 $B_{1}^{+}$-inhomogeneity corrected maps: UNI, so-called “denoised” UNI (UNIDEN), and qT_1_ maps using the same previous in-house scripts and relaxation model extended to a bijective 3D lookup-table (UNI, $B_{1}^{+}$, T_1_).

Brain masks were generated from the UNIDEN maps and using FreeSurfer’s^55^ *mri_synthstrip*^56^. UNI maps were masked using their associated UNIDEN’s brain mask and FMRIB Software Library’s^57^ (FSL) *fslmaths*. Brain segmentations & parcellations were generated using masked UNI maps and FreeSurfer’s *mri_synthseg*^58,59^ (v2 & robust mode). The MNI152 ICBM 2009c Nonlinear Symmetric^60,61^ T_1_-weighted template was masked using FreeSurfer’s *mri_synthstrip* and nonlinear-registered onto the UNI maps using ANTs’ *antsRegistration*. The template’s white matter (WM) probability map and lobe atlas^28^ were resampled and nonlinear-registered onto the UNI maps using the warp field from the previous registration and ANTs’ *antsApplyTransforms*.

Every MT volume was acquired with at least 5 s of dummy repetitions. MT pulses were turned off after 50 % of the k-space lines were acquired on the phase/partition 2D plane. The k-space was subsequently sampled using a center-out cartesian spiral^23^ trajectory.

For each volunteer, all MT volumes sharing the same readout module parameters were concatenated together using FSL’s *fslmerge*. The concatenated 3D volumes were denoised using a conda^62,63^ environment with Python^64^ and the MP-PCA algorithm implemented in MRtrix3’s^65^ *dwidenoise*^66–68^. The volumes were then apodized using a cosine window function^25^, zero-filled to twice their original size in all 3D direction, and motion-corrected as described in Soustelle L. (2020^29^, 2022^24^), moving every concatenated MT volume to a singular MT space. Finally, they were split back into a set of 3D MT volumes using FSL’s *fslsplit*. Then, MT ratio^30^ (MTR) and ihMT ratio^26^ (ihMTR) maps were computed as

$$ihMTR=\frac{ihMT}{MT_{0}}=\frac{MT_{+}^{s}+MT_{-}^{s}-MT_{\pm}^{d}-MT_{\mp}^{d}}{MT_{0}}=M{TR}_{\pm}^{d}+M{TR}_{\mp}^{d}-M{TR}_{+}^{s}-M{TR}_{-}^{s}$$

$MTR \& ihMTR$ values outside of the $[0, 1]$ range were correlated to poor SNR and were set to 0. A second processing stage, for SNR quantification purposes, was performed on the preprocessed MT volumes using the same previous steps – barring MP-PCA, cosine-apodization, and 0-filling – to compute ihMT maps. $ihMT<0$ values were correlated to poor SNR and were set to 0.

For each volunteer, for each MT space, a reference ihMT map was computed, masked using FreeSurfer’s *mri_synthstrip*, and rigid-registered onto the volunteer’s masked UNI map using ANTs’ *antsRegistration*. Then, the qT_1_, brain mask, segmentations & parcellations, atlases, and $B_{1}^{+}$ field map were resampled and registered from UNI space to MT space using the inverse transformation matrix of the ihMT registration and ANTs’ *antsApplyTransforms*.

In both Experiment A & Experiment B, each (ih)MTR volume was masked by the cerebral WM segmentation and a mask of the $B_{1}^{+}$ field map such that only (ih)MTR values associated with a relative $B_{1}^{+}$ within a 2.5 % margin from the nominal value were kept ($B_{1,rel}^{+}=100 \%\pm2.5 \%$, with $B_{1,rel}^{+}=100 \%$ the nominal field intensity). Resulting (ih)MTR distributions were independently fitted as skew-logistic^31^ distributions using the same previous conda environment & Python, along with SciPy’s^69^ *stats.genlogistic.fit* with location and scale parameters left free. (ih)MTR modes, means, and standard deviations were extracted from the fits in lieu of using data means & standard deviations.

Moreover, each ihMT volume was masked by the cerebral WM segmentation of FreeSurfer’s *mri_synthseg*, the MNI152 lobe atlas, a mask of the MNI152 WM probability atlas above of values above 95 %, and a mask of the $B_{1}^{+}$ field map such that only (ih)MTR values associated to $B_{1,rel}^{+}=100 \%\pm2.5 \%$ were kept, resulting in 8 Regions of Interest (ROI): left & right frontal, temporal, occipital, and parietal cerebral WM that were then combined together. Resulting ihMT distributions were assumed Gaussian and were independently fitted using the same previous conda environment & Python, along with SciPy’s *stats.norm.fit* with location and scale parameters left free. ihMT means and standard deviations were extracted from the data.

Brain slices shown in this paper were generated from screen captures of ITK-Snap^70^ (Fig. 4, 8, 9-a, 10) and FreeView (Fig. 9-b) windows, or as output of Python’s library Matplotlib’s^71,72^ *imshow* function (Fig. 7) from the same previous conda environment.

## Bloch-McConnell-Solomon-Provotorov Equations for ihMT modeling

$$\frac{\partial M_{x}^{A}}{\partial t}=-\frac{M_{x}^{A}}{T_{2}^{A}}+\gamma\left( \vec{M}^{A}\times\vec{B}_{1} \right)\cdot\vec{e}_{x}+\Delta\omega\cdot M_{y}^{A}$$

$$\frac{\partial M_{y}^{A}}{\partial t}=-\frac{M_{y}^{A}}{T_{2}^{A}}+\gamma\left( \vec{M}^{A}\times\vec{B}_{1} \right)\cdot\vec{e}_{y}-{\Delta\omega\cdot M}_{x}^{A}$$

$$\frac{\partial M_{z}^{A}}{\partial t}=\frac{M_{0}^{A}-M_{z}^{A}}{T_{1}^{A}}+\gamma\left( \vec{M}^{A}\times\vec{B}_{1} \right)\cdot\vec{e}_{z}+R\left( {M_{0}^{A}M}_{z}^{B}-{M_{0}^{B}M}_{z}^{A} \right)$$

$$\frac{\partial M_{z}^{B}}{\partial t}=\frac{\left( M_{0}^{B}-M_{z}^{B} \right)}{T_{1}^{B}}+{\frac{\Delta\omega}{\left| \Delta\omega\right|}\pi\left( \gamma\vec{B}_{1} \right)^{2}g_{SL}(\Delta\omega,T_{2}^{B})\times(M_{D}^{B}-M}_{z}^{B})-R\left( {M_{0}^{A}M}_{z}^{B}-{M_{0}^{B}M}_{z}^{A} \right)$$

$$\frac{\partial M_{D}^{B}}{\partial t}=-\frac{M_{D}^{B}}{T_{1}^{D}}-\frac{\Delta\omega}{\left| \Delta\omega\right|}\pi\left( \gamma\vec{B}_{1} \right)^{2}g_{SL}(\Delta\omega,T_{2}^{B})\times(M_{D}^{B}-M_{z}^{B})\left( \frac{\Delta\omega}{\omega_{D}} \right)^{2}$$

The transverse magnetization of the water proton pool $\left[ M_{x}^{A}, M_{y}^{A} \right]$ is considered perfectly spoiled after every RF pulse and is set to 0 after every RF pulse in our numerical simulations. $M_{D}^{B}=\Delta\omega\cdot\beta$ with $\beta$ the inverse dipolar spin temperature such that $M_{D}^{B}$ has the same dimensionality as $M_{Z}^{B}$. The coupling between $M_{Z}^{B}$ & $M_{D}^{B}$ is set to 0 when performing dual offset saturation symmetrically around on-resonance. $g_{SL}(\Delta\omega, T_{2}^{B})$ is the super-Lorentzian absorption lineshape describing the non-aqueous “semi-solid” pool. $\Delta\omega$ is the RF saturation frequency and is variable in time. $\omega_{D}$ is the local average dipolar field strength. $\gamma$ is the gyromagnetic ratio of water hydrogen protons. $\vec{B}_{1}$ is the magnetic flux density of the RF saturation field and is variable in time.

## Model fitting

For the purpose of SNR efficiency optimization and generation of a suitable $B_{1}^{+}$-correction law, we made use of the standard binary spin-bath or two-pool model (2-PM)^73–75^, consisting of an aqueous “mobile” pool A and non-aqueous “semi-solid” pool B. As previously motivated, the non-aqueous reservoir was equipped with an additional dipolar reservoir to account for the observed differences in single- and dual-sided saturation according to the Provotorov theory^76^. Of note, the dipolar reservoir (described by an inverse spin temperature $\beta$) is uncoupled from the non-aqueous reservoir in the absence of RF irradiation. In total, this 2-PM is described by a set of 8 biophysical parameters ($T_{1}^{A}$, $T_{2}^{A}$, $T_{1}^{B}$, $T_{2}^{B}$, between-pool exchange rate k, MPF, $T_{1}^{D}$, $\omega_{D}$). Furthermore, a parameter $\delta f_{mm}^{B}$ was introduced to describe the frequency shift of the underlying broad spectrum of non-aqueous protons relative to the water resonance. Due to the limited sensitivity of the experimental data acquired in this study to certain model parameters, some of them were set to appropriate values (Fig. 3). For a super-Lorentzian absorption lineshape, the local average dipolar field strength $\omega_{D}$ (in radians per second) can be calculated as^75^

$$\omega_{D}=\frac{1}{\sqrt{15} T_{2}^{B}}$$

An established matrix-algebra approach implemented in MATLAB^77^, based on the modified Bloch-McConnel equations^78^ as described in detail elsewhere^32,79,80^, was used to simulate the evolution of the magnetization throughout the pulse sequences. Briefly, RF pulses were approximated by piece-wise constant RF parameters for discrete timesteps ($\Delta t_{i}=20$ μs). For the non-aqueous pool, a super-Lorentzian RF absorption lineshape function $g_{SL}$ was assumed to calculate the off-resonant saturation rate

$$R_{RF}^{B} ({\Delta f}_{RF}, T_{2}^{B})=\pi\omega_{1}^{2}g_{SL}({\Delta f}_{RF},T_{2}^{B})$$

Any transverse magnetization of non-aqueous protons was neglected, as is common practice, due to its rapid loss of coherences^73,81^. Perfect spoiling was assumed for the transverse magnetization of aqueous protons. The sequence simulation also includes all the small rectangular pulses ($FA = 5$ °, $pw = 100$ $\mu s$) of the imaging module under the simplified assumption that the effect of these pulses on the non-aqueous is negligibly small.

The estimated modes of the distributions (Section 11 of Supplementary Materials, Table S2 & S3) for the four computed MT ratios and the corresponding ihMT ratios of all cerebral white matter regions, characterized by nominal $B_{1}^{+}$ values of 100 % ± 2.5 %, were fitted jointly for the three subjects of the model training dataset. During the fitting procedure, based on MATLAB’s non-linear least-square estimator *lsqcurvefit*, the remaining five free model parameters were adjusted. Confidence intervals were computed using MATLAB’s *nlparci* function.

## Samplings of the fitted model parameters in Monte-Carlo uncertainty analysis


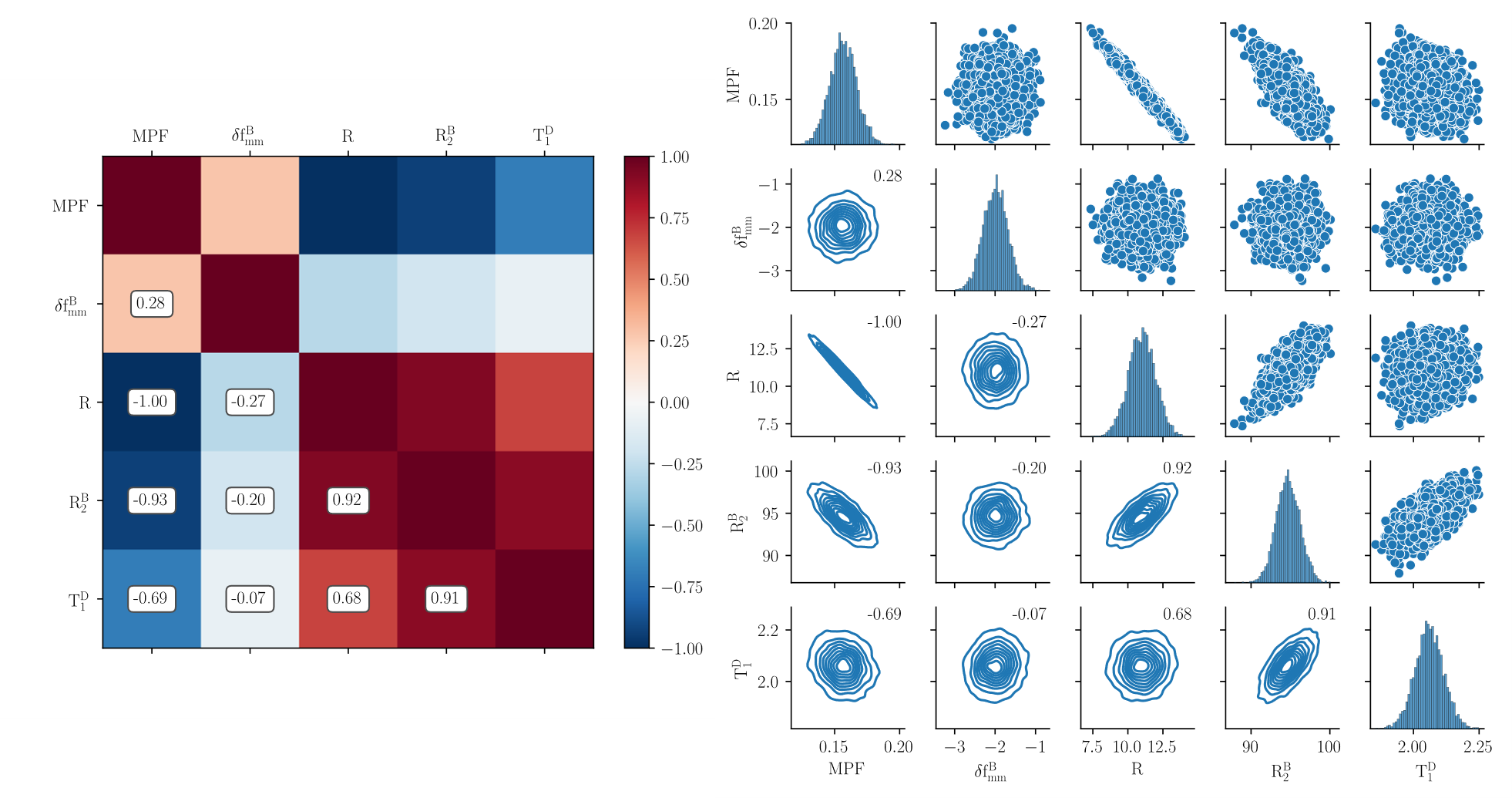


Figure S2. (left) correlation matrix of the fitted parameters, (right) sampled model parameters using SciPy's multivariate_normal function and the covariance matrix of the fitted parameters for sample generation and Matplotlib’s matshow function and Seaborn’s PairGrid function for visualization of the correlation matrix and distribution samplings respectively. Seaborn’s PairGrid function uses the standard kernel density estimation, scatter, and histogram plots with default parameters.

## Saturation module optimization

To study the optimal SNR efficiency regime for the ihMT signal, we simulated varying parametrization of the SAR-Efficient ihMT sequence for ALT and CM saturation modules, with minimal TR such that the sequence is estimated to fit within SAR regulations. Because the SNR of an $\mathrm{ihMTR}$ image is small (~10 %) compared to the SNR of an $MT_{0}$, we can trivially show using log-expansion that the SNR of $\mathrm{ihMTR}$ is approximately equal to the SNR of $\mathrm{ihMT}$. We have thus used the SNR of ihMT as a proxy for the SNR of ihMTR. Finally, to stay within time constraints of a clinical research protocol ($\leq2$ h), any sequence with $\mathrm{TR}\geq2100$ ms were deemed too long and not acquired, as well as any sequence deemed too inefficient, such as the cosine-modulated sequences with more than 1 pulse or less than 3 bursts and the frequency-alternated sequences with less than 4 pulses (except ALT 3B2P) or less than 3 bursts.

### Cosine-modulated saturation optimization


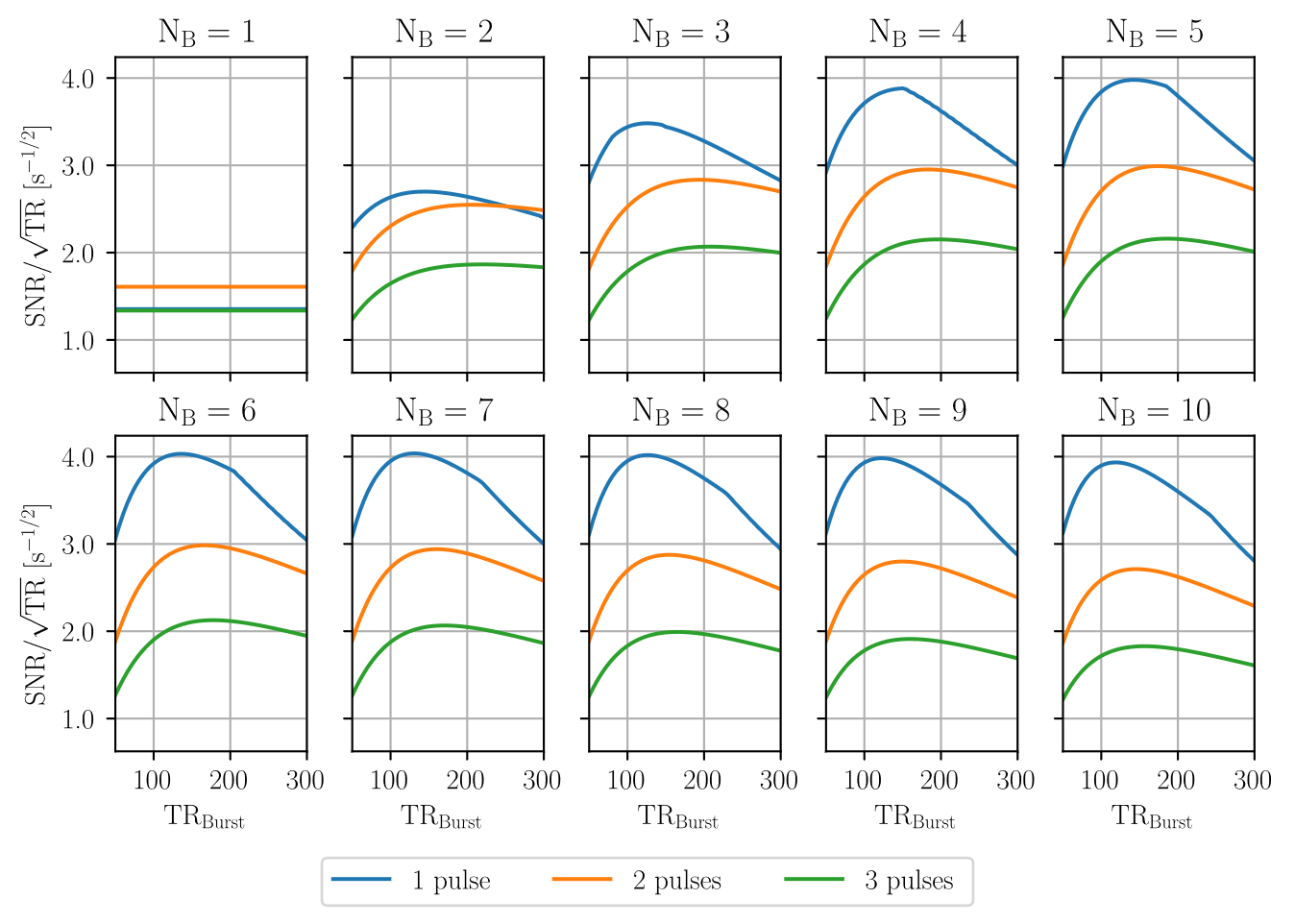


Figure S3. Cosine modulated saturation module simulations for the optimization of $SNR/\sqrt{TR}$ depending on the number of pulses $N_{P}$ and $TR_{Burst}$ within a figure and the number of bursts $N_{B}$ across figures.


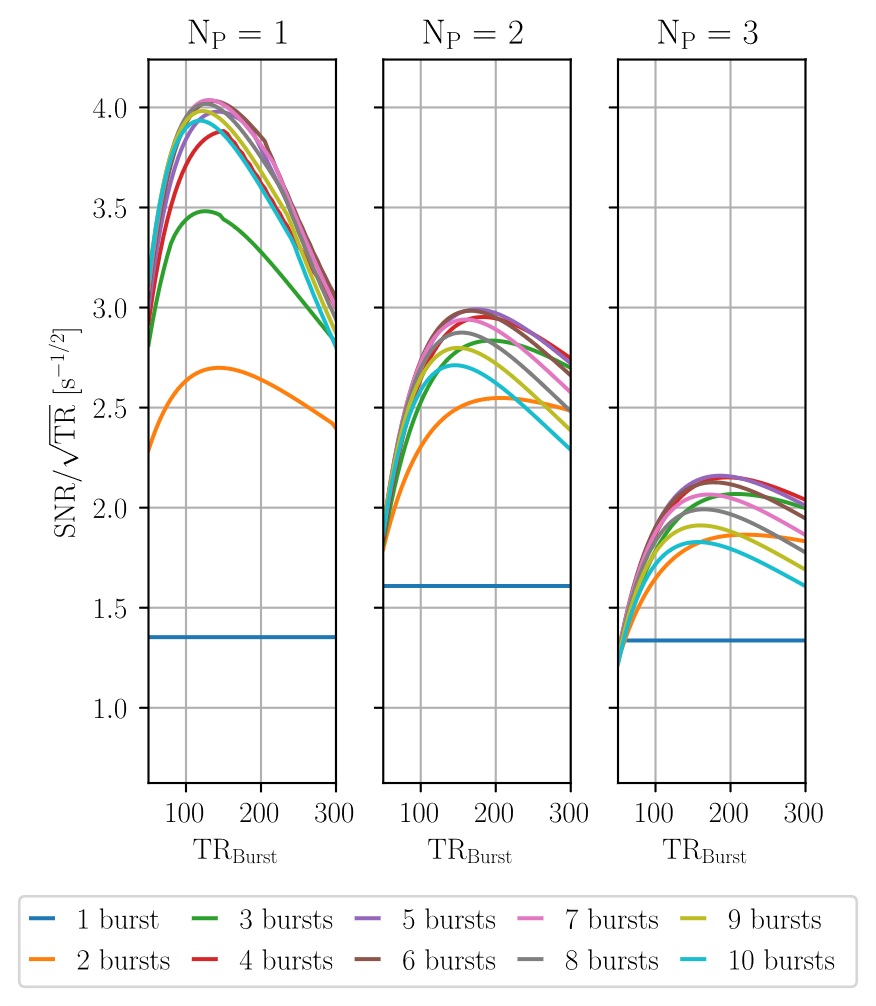


Figure S4. Cosine-modulated saturation module simulations for the optimization of $SNR/\sqrt{TR}$ depending on the number of bursts $N_{B}$ and $TR_{Burst}$ within a figure and the number of pulses $N_{P}$ across figures.


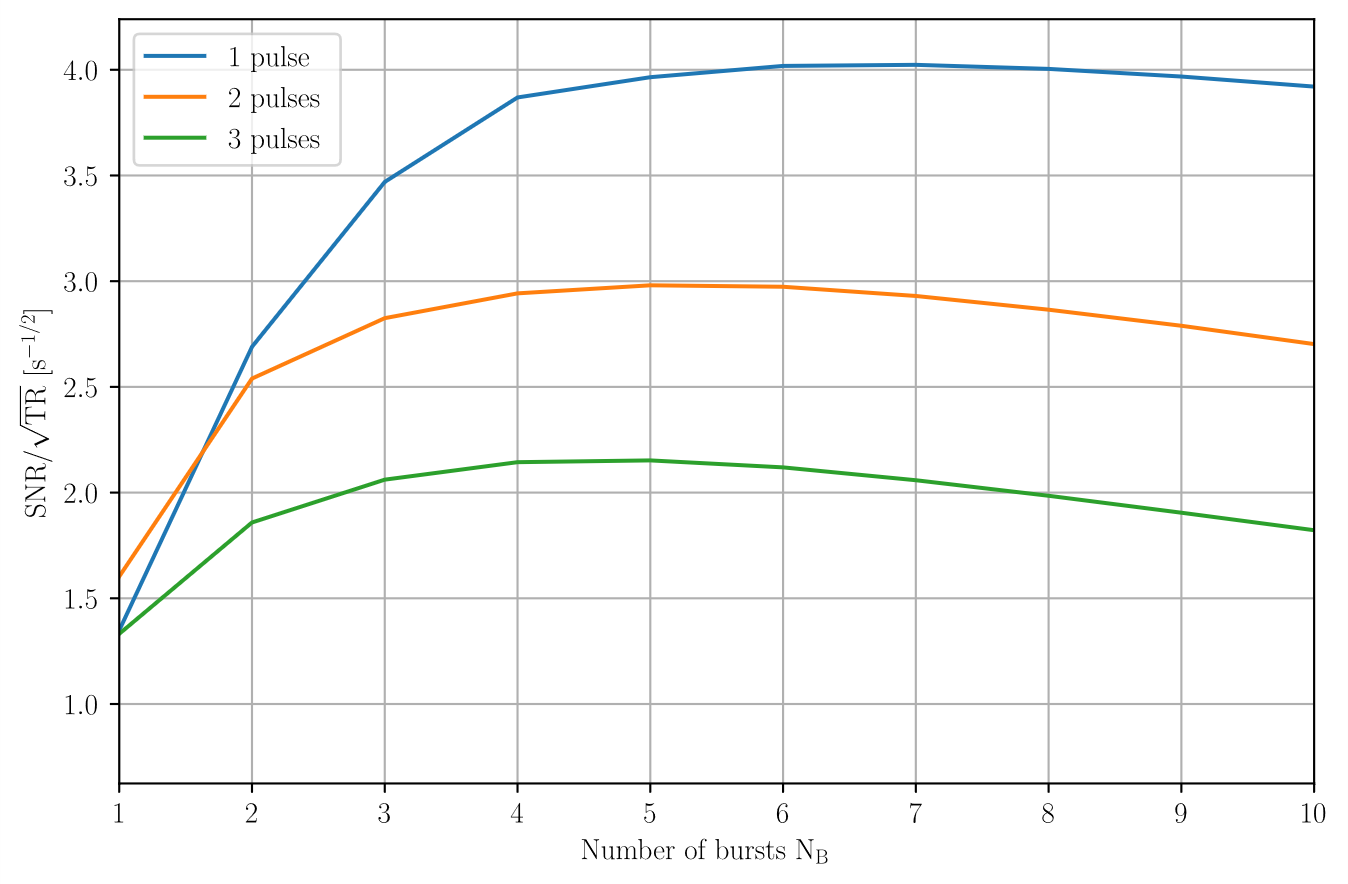


Figure S5. Cosine-modulated saturation module simulations for the optimization of $SNR/\sqrt{TR}$ depending on the number of bursts $N_{B}$ and number of pulses $N_{P}$ given an optimal $TR_{Burst}$.

### Frequency-alternated saturation optimization


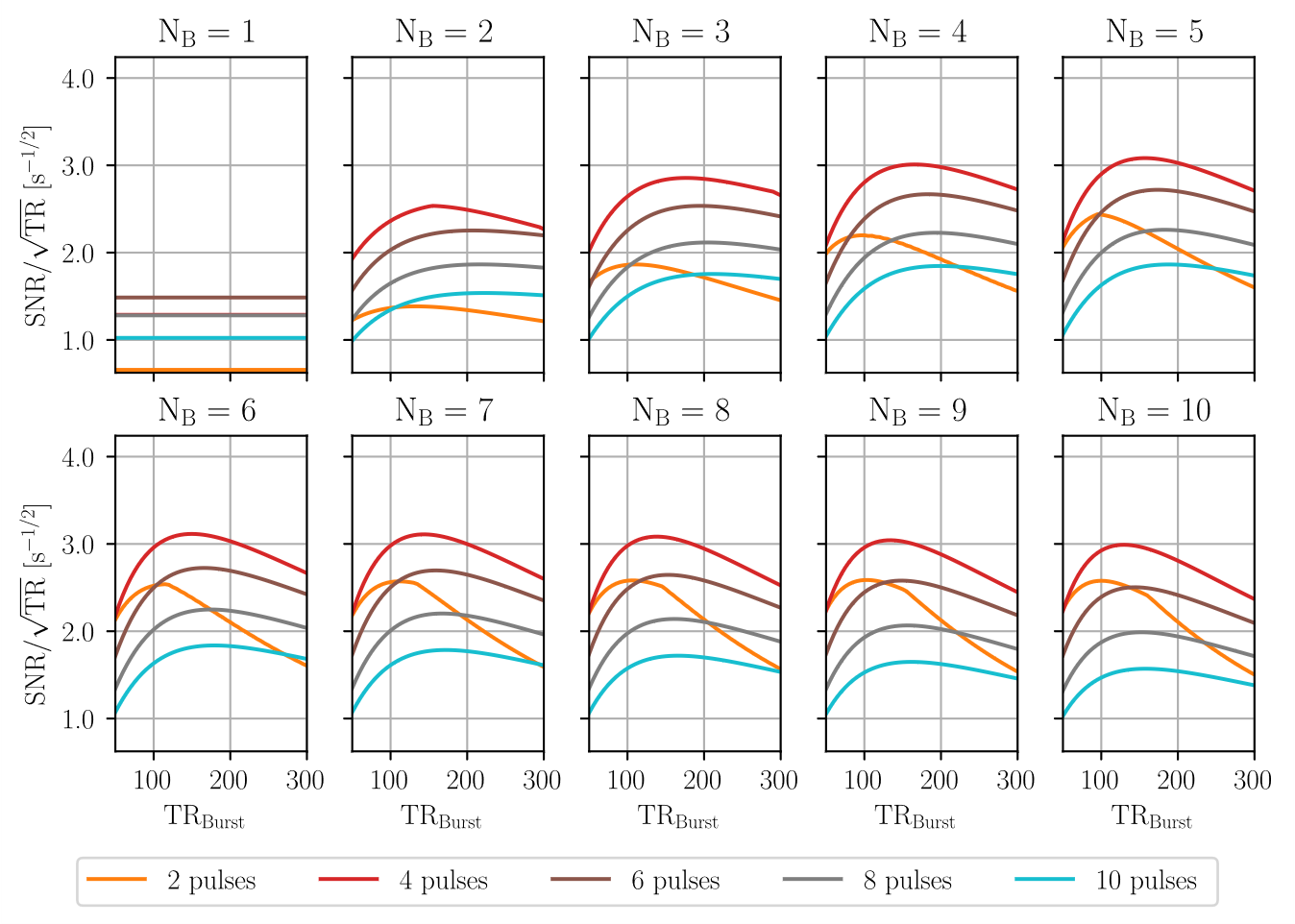


Figure S6. Frequency-alternated saturation module simulations for the optimization of $SNR/\sqrt{TR}$ depending on the number of pulses $N_{P}$ and $TR_{Burst}$ within a figure and the number of bursts $N_{B}$ across figures.


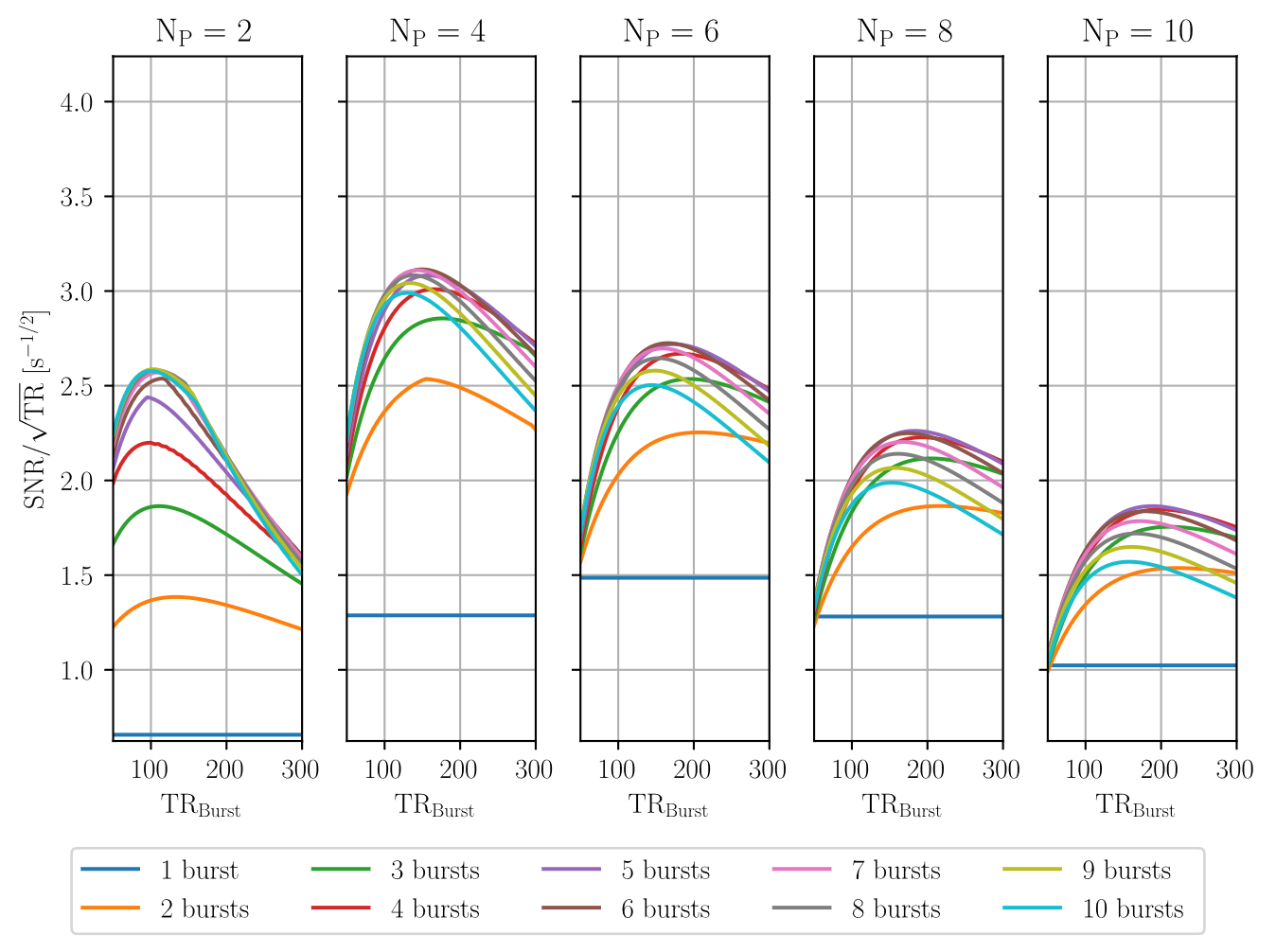


Figure S7. Frequency-alternated saturation module simulations for the optimization of $SNR/\sqrt{TR}$ depending on the number of bursts $N_{B}$ and $TR_{Burst}$ within a figure and the number of pulses $N_{P}$ across figures.


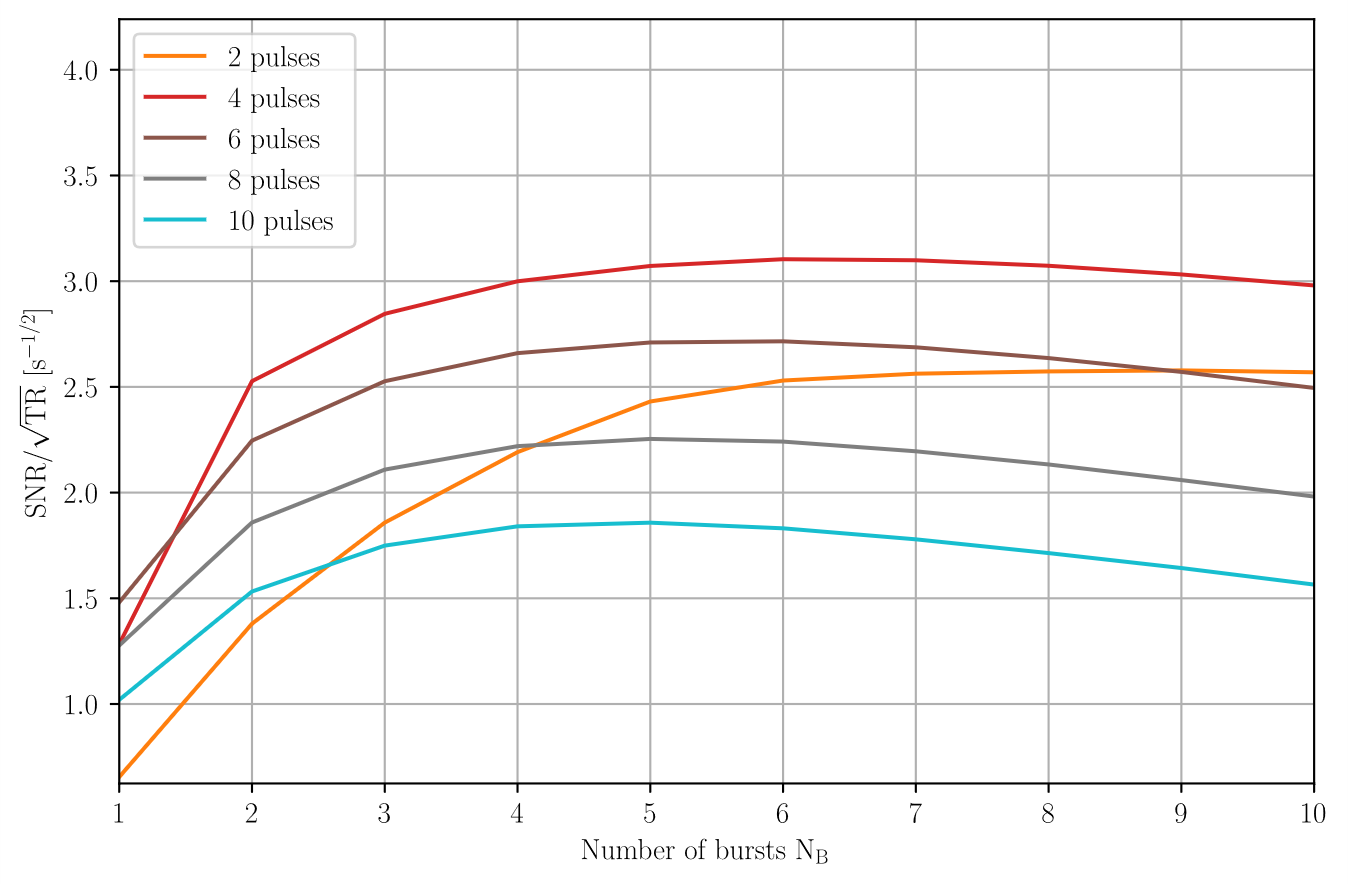


Figure S8. Frequency-alternated saturation module simulations for the optimization of $SNR/\sqrt{TR}$ depending on the number of bursts $N_{B}$ and number of pulses $N_{P}$ given an optimal $TR_{Burst}$.


## $B_{1}^{+}$ corrections

Forward simulations were performed based on the estimated model parameters and different nominal $B_{1}^{+}$ scaling factors for each sequence (varying from 15 % to 130 % using 2.5 % increments). Afterwards, $n_{rdm}=5000$ forward simulations were performed with varying model parameters at all nominal $B_{1}^{+}$ scaling factors for each sequence. The model parameters were generated using SciPy’s *multivariate_normal* function and *lsqcurvefit*’s output covariance matrix (see Supplementary Materials Sections 5 & 6). The resulting discretized $B_{1}^{+}$ dependencies and variation bands were interpolated in Python using SciPy’s *PchipInterpolator* function to create the continuous $B_{1}^{+}$ corrections shown in Figure 6.

## Unbiased estimator of the population intra & inter-individual coefficients of variation of a normal-distributed random variable

In the case of a normal-distributed random variable $X \sim\mathcal{N}\left( \mu, \sigma\right)$, while the sample expectation $E[X]$ & variance $Var\left[ X \right]$ are unbiased estimators of the population expectation $\mu$ & variance $\sigma^{2}$, the sample standard deviation $STD\left[ X \right]$ is not an unbiased estimator of the population standard deviation $\sigma$^54^. However, it is possible to retrieve an unbiased estimation of the population standard deviation by using the correction $c_{4}(n)$ defined as such^34^

$$c_{4}\left( n \right)=\sqrt{\frac{2}{n-1}}\frac{\boldsymbol{\Gamma}\left( \frac{n}{2} \right)}{\boldsymbol{\Gamma}\left( \frac{n-1}{2} \right)}, \mathrm{which} \left\{ \begin{aligned} {\mathrm{for} n=2: c}_{4}\left( 2 \right)= \sqrt{\frac{2}{\pi}}\approx0.79788 [1] \\ {\mathrm{for} n=4: c}_{4}\left( 4 \right)= 2\sqrt{\frac{2}{3\pi}}\approx0.92132 [2] \end{aligned} \right.$$

where $\boldsymbol{\Gamma}\left( \cdot\right)$ is the gamma function and $n$ is the number of samples. The unbiased estimator of the population standard deviation $\sigma^{*}$ is given by^34^

$$\sigma^{*}=\frac{STD[X]}{c_{4}\left( n \right)} [3]$$

Let ihMTR (resp. SNR) values be drawn from a generic distribution such that the Central Limit Theorem^84^ applies. The sample expectation $E[ihMTR]=X \sim N\left( \mu,\sigma\right)$ (resp. $E[SNR]=X \sim N\left( \mu,\sigma\right)$) is a normal-distributed random variable.

Using Eq. 1 & 3, the unbiased estimator of the population intra-individual coefficient of variation for a given individual $i$ and sequence $s$ in Experiment A is

$${CV}_{i,s}^{intra}=\frac{\sigma_{i,s}^{*, intra}}{{E\left[ X \right]}_{i,s}^{intra}}=\frac{1}{c_{4}\left( 2 \right)}\frac{{STD\left[ X \right]}_{i,s}^{intra}}{{E\left[ X \right]}_{i,s}^{intra}}=\sqrt{\frac{\pi}{2}}\frac{{STD\left[ X \right]}_{i,s}^{intra}}{{E\left[ X \right]}_{i,s}^{intra}} \left[ 4 \right]$$

$$=\sqrt{\frac{\pi}{2}}\frac{\sqrt{\frac{1}{(2-1)}\sum_{j=1}^{2} \left( {E[X]}_{j,i,s}-{E\left[ X \right]}_{i,s}^{intra} \right)^{2}}}{{E\left[ X \right]}_{i,s}^{intra}} [5]$$

where ${E\left[ X \right]}_{i,s}^{intra}$ and $\sigma_{i,s}^{*,intra}$ are the unbiased estimators of the population expectation & standard deviation of the mean ihMTR (resp. SNR) from all repetitions $j$ given an individual $i$ and a sequence $s$ in Experiment A. Here, we only perform 2 repetitions (test-retest), therefore $j\in\{1, 2\}$.

Finally, using Eq. 2 & 3, the unbiased estimator of the population inter-individual coefficient of variation for a given sequence $s$ in Experiment A is

$$CV_{s}^{inter}=\frac{\sigma_{s}^{*,inter}}{{E\left[ X \right]}_{s}^{inter}}=\frac{1}{c_{4}\left( 4 \right)}\frac{{STD\left[ X \right]}_{s}^{inter}}{{E\left[ X \right]}_{s}^{inter}}=\sqrt{\frac{3\pi}{8}}\frac{{STD\left[ X \right]}_{s}^{inter}}{{E\left[ X \right]}_{s}^{inter}} \left[ 6 \right]$$

$$=\sqrt{\frac{3\pi}{8}}\frac{\sqrt{\frac{1}{\left( 4-1 \right)}\sum_{i=1}^{4} \left( {E\left[ X \right]}_{i,s}^{intra}-{E\left[ X \right]}_{s}^{inter} \right)^{2}}}{{E\left[ X \right]}_{s}^{intra}} [7]$$

where ${E\left[ X \right]}_{s}^{inter}$ and $\sigma_{s}^{*,inter}$ are the unbiased estimators of the population expectation & standard deviation of the mean ihMTR (resp. SNR) from all individuals $i$ for a given sequence $s$ in Experiment A. Here, we only perform the Experiments on 4 volunteers, therefore $i\in\{1, 2, 3, 4\}$.

## Thermal noise estimates and SNR quantification


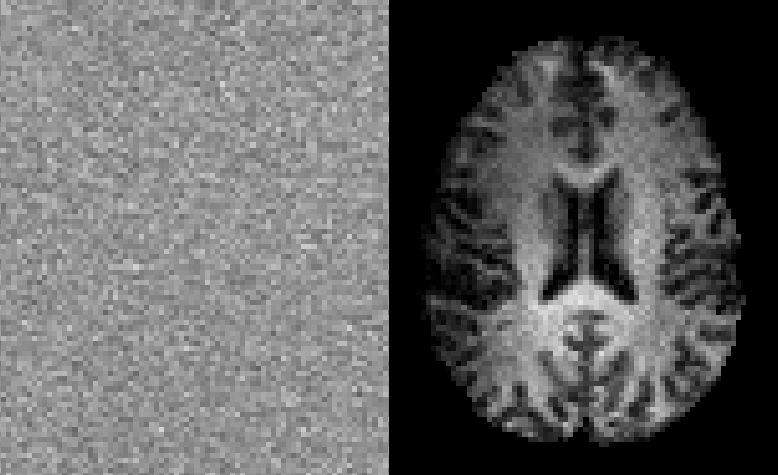


Thermal Noise

ihMT

Figure S9. Acquired thermal noise map (left) and the associated minimally-processed ihMT map (right) for the ihMTR CM5B1P at 2.0 mm iso.

Computation of SNRs were done in image space. A thermal noise map was acquired for each different ihMT acquisition readout (e.g., change in resolution, FOV, number of readout segment per TR, etc.). To acquire the thermal noise map, an $MT_{0}$ mapping sequence associated with the ihMT acquisition was run, setting the reference coil load to $V_{ref}=0 \%$, such that only the thermal noise is acquired during Analog-to-Digital Converter segments. Noise maps were then reconstructed using the same GRAPPA reconstruction algorithm as with ihMT maps.

Because GRAPPA reconstruction requires k-space calibration lines to regularize its algorithm, it was not certain that this method of estimating thermal noise properties was stable. As such, we repeated the same acquisition 3 times and verified that all thermal noise maps had similar arithmetic means and standard deviations, which confirmed that this thermal noise estimation method was sufficiently robust.

## Collections of quantitative metrics for the various ihMTR acquisitions of this study


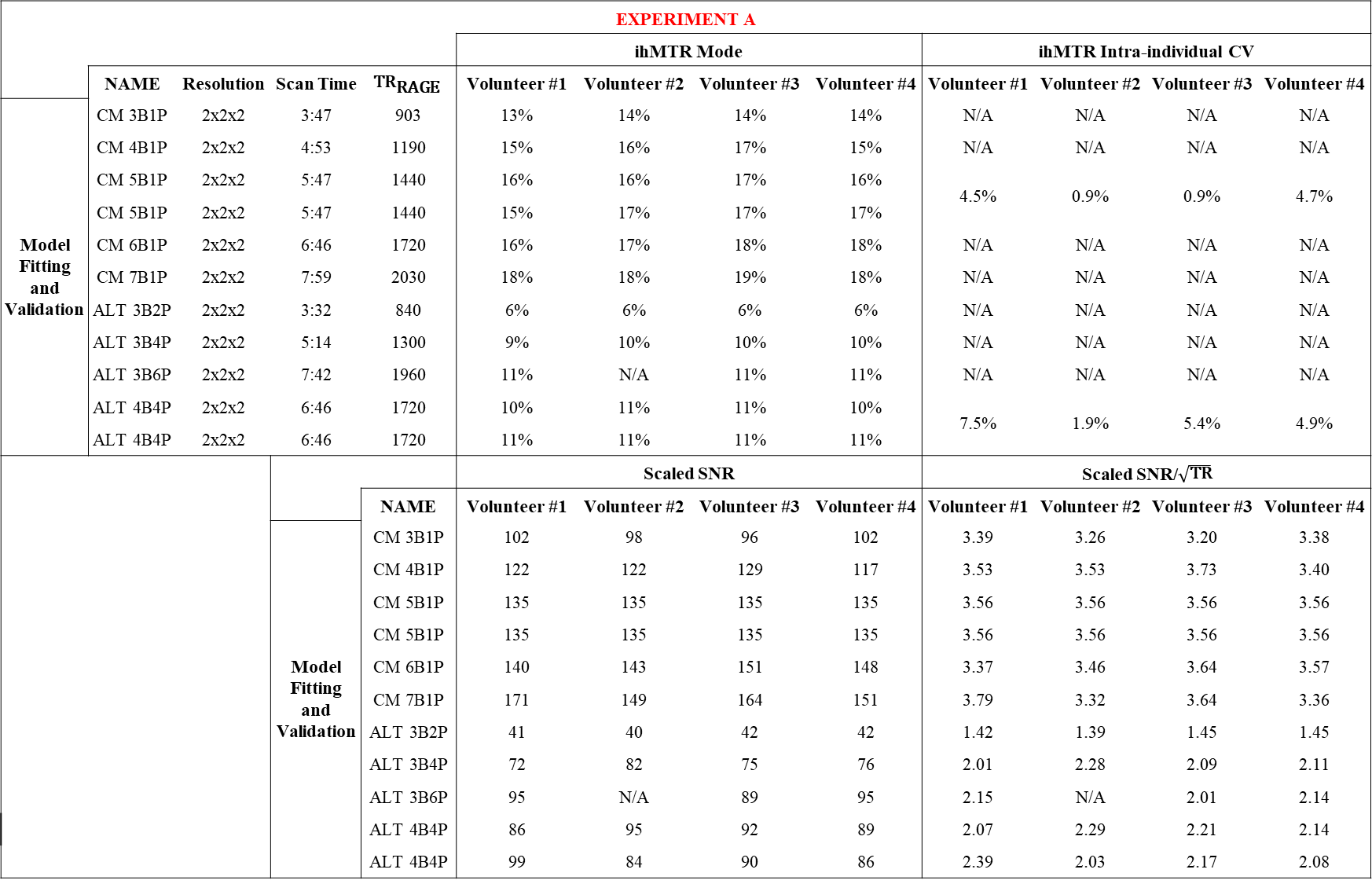


Table S2. Quantitative metrics (ihMTR & SNR) for Experiment A.

| **EXPERIMENT B** |  |  |  |  |  |  |  |  |
| --- | --- | --- | --- | --- | --- | --- | --- | --- |
|  | **NAME** | **Resolution** | **Scan Time** | $\text{T}\text{R}_{\text{RAGE}}$ | **ihMTR mode** | **SNR** | **SNR/**$\sqrt{\text{TR}}$ | $\text{V}_{\text{ref}}$ |
| **Variable** $\text{V}_{\text{ref}}$ | CM 5B1P | 2x2x2 | 5:47 | 1440 | 16% | 135 | 3.56 | 100% |
|  | CM 5B1P | 2x2x2 | 5:47 | 1440 | 13% | 95 | 2.51 | 80% |
|  | CM 5B1P | 2x2x2 | 5:47 | 1440 | 8% | 47 | 1.23 | 60% |
|  | CM 5B1P | 2x2x2 | 5:47 | 1440 | 5% | 20 | 0.51 | 40% |
| **Full FOV High Resolution** | CM 5B1P | 1.4x1.4x1.4 | 10:14 | 1440 | 15% | 76 | 1.99 | N/A |
|  | CM 5B1P | 1.2x1.2x1.2 | 12:38 | 1440 | 16% | 54 | 1.41 | N/A |
| **Reduced FOV High Resolution** | CM 5B1P | 1.2x1.2x1.2 | 5:47 | 1440 | 16% | 34 | 0.91 | N/A |
|  | CM 5B1P | 1x1x1 | 7:57 | 1440 | 17% | 25 | 0.65 | N/A |

Table S3. Quantitative metrics (ihMTR & SNR) for Experiment B.

## MT and MTR images from a representative whole-brain ihMT acquisition (CM 5B1P) at 1.2 mm iso.


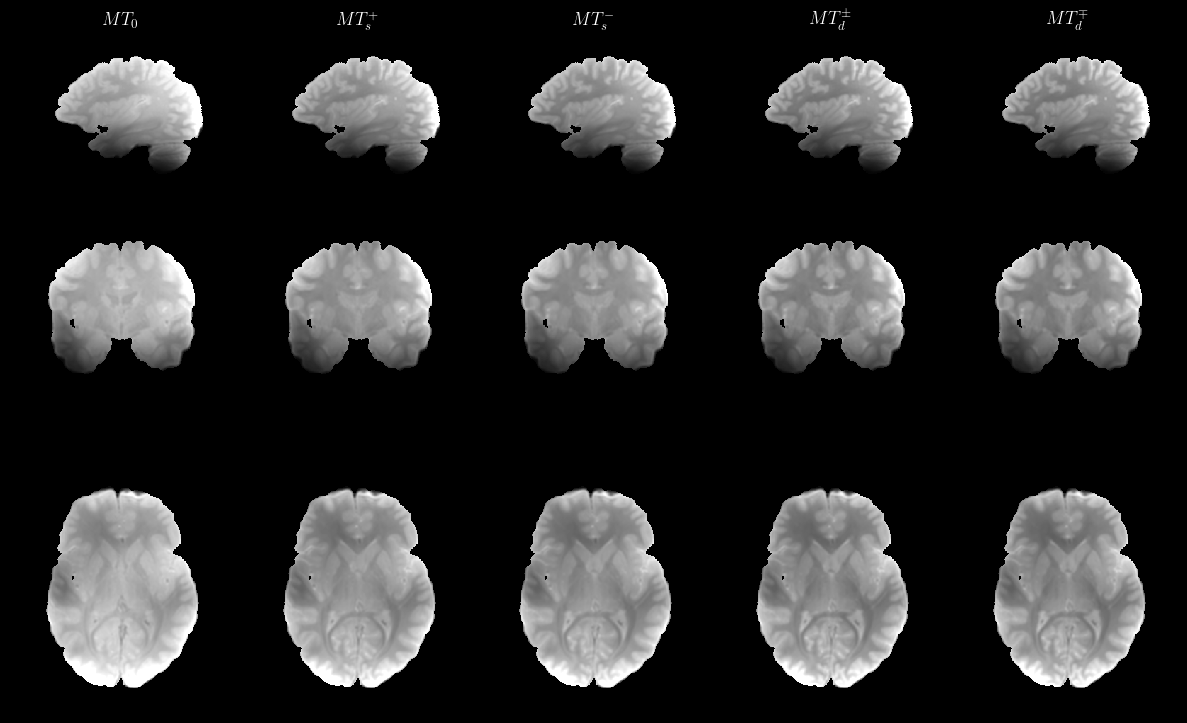


Figure S10: MT images of whole-brain CM 5B1P at 1.2 mm iso. in arbitrary DICOM scale, all within the same range. No $B_{1}^{+}$ correction was applied.


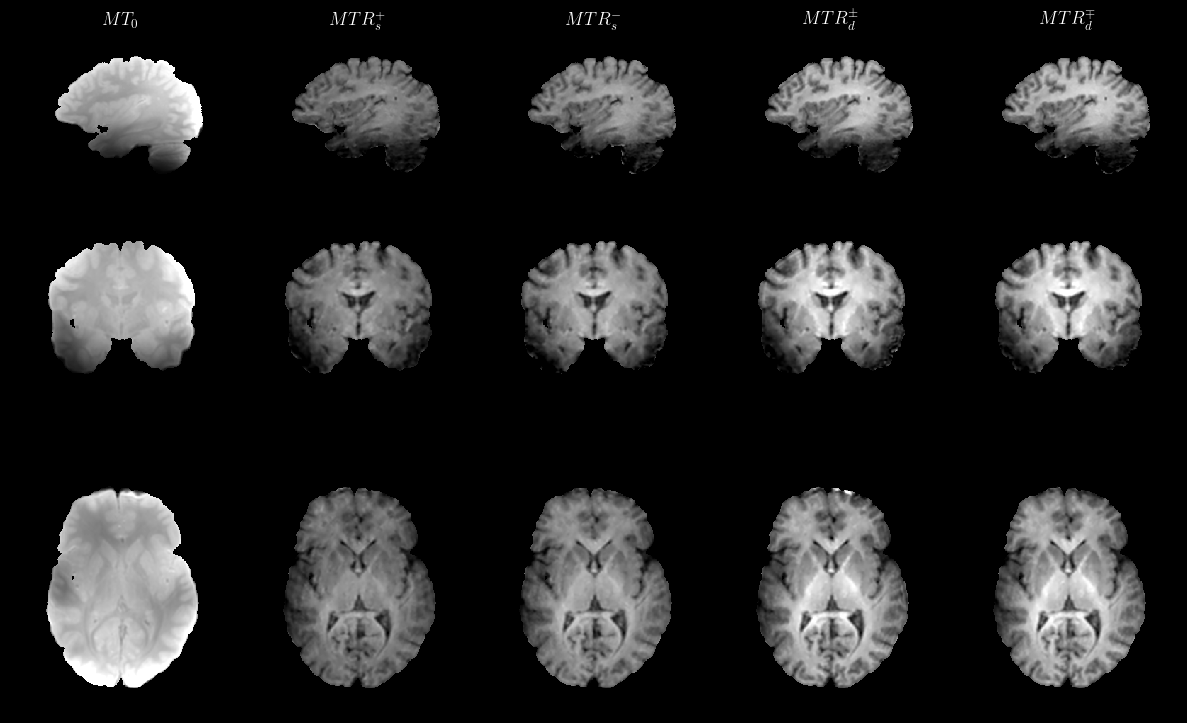


Figure S11: MTR images of whole-brain CM 5B1P at 1.2 mm iso. (MT_0) in arbitrary DICOM scale,(MTR) scaled from [0, 60] %. No $B_{1}^{+}$ correction was applied.

## Line profiles as qualitative proxy for contrast sharpness quantification

### Line profile of MT images and MTR for a single whole-brain ihMT acquisition (CM 5B1P at 1.2 mm iso.)

#### MT line profiles


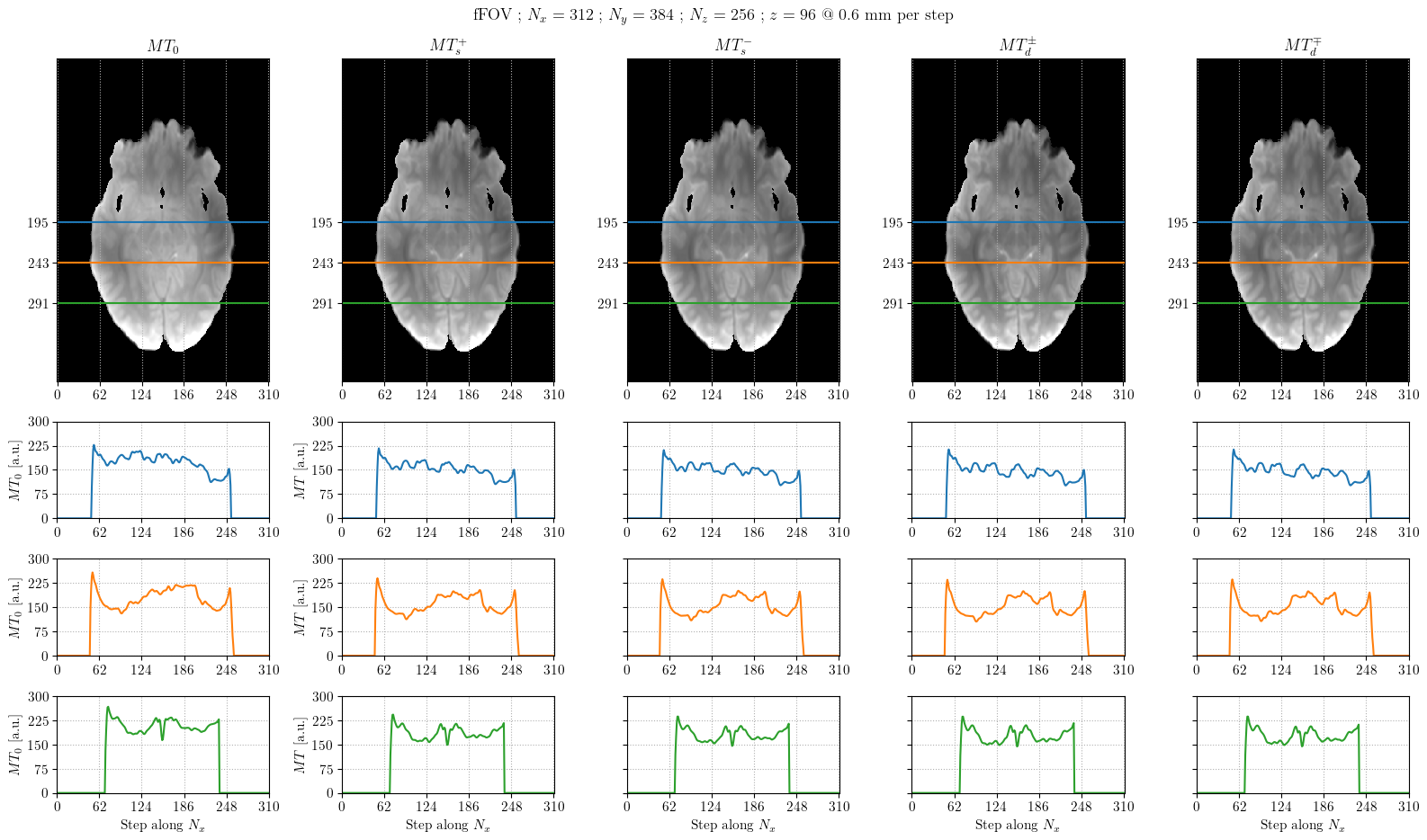


Figure S12: Line profiles of CSF-masked MT images for the CM 5B1P 1.2 mm iso. whole-brain sequence. From left to right, contrasts are $MT_{0}$, $MT_{s}^{+}$, $MT_{s}^{-}$, $MT_{d}^{\pm}$, $MT_{d}^{\mp}$. Visually, the images shown in the top row may be stretched vertically and horizontally, fitting the graph frames to make comparison between columns more straightforward.

#### MTR line profiles


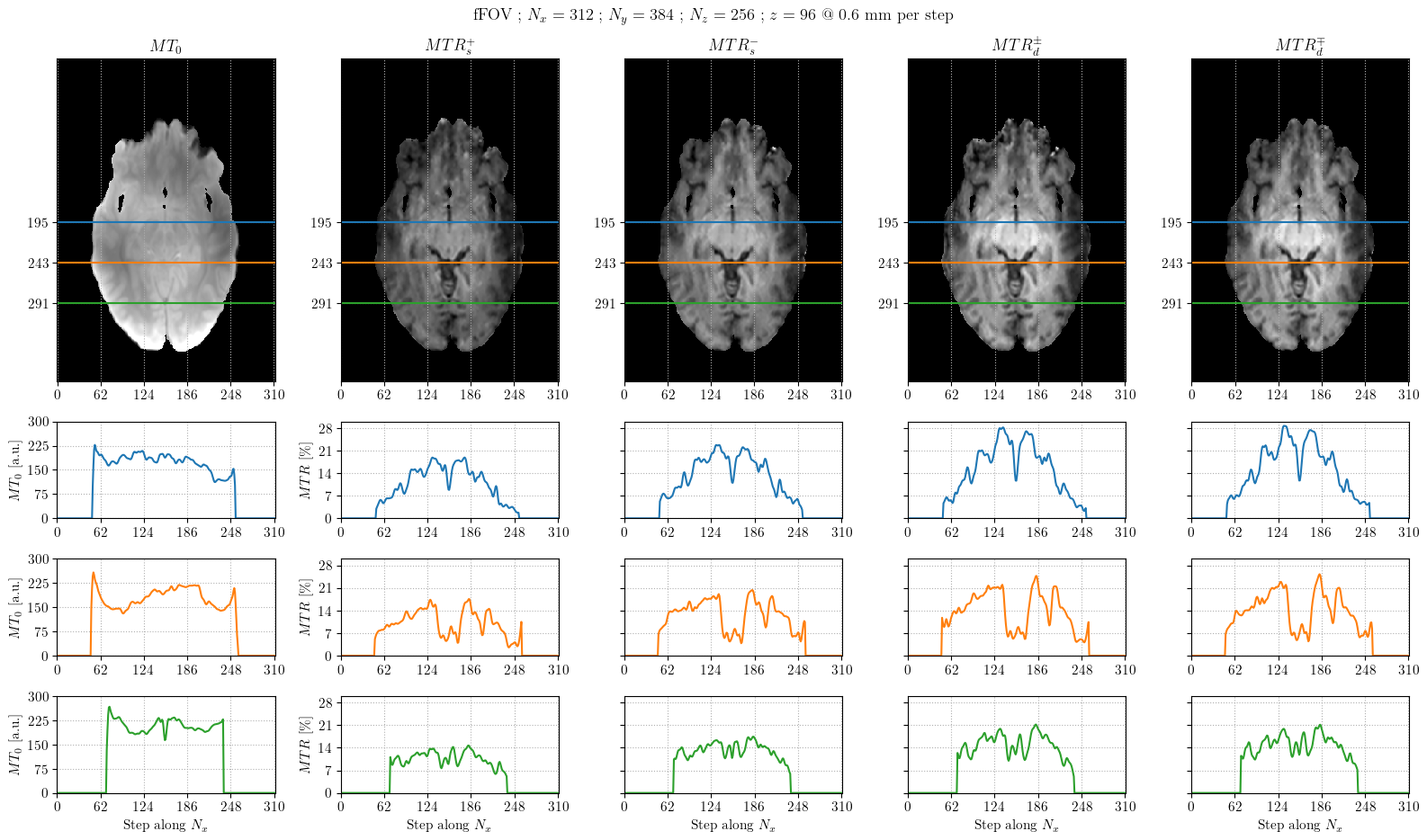


Figure S13: Line profiles of CSF-masked MTR images for the CM 5B1P 1.2 mm iso. whole-brain sequence. From left to right, contrasts are $MT_{0}$, $M{TR}_{s}^{+}$, $M{TR}_{s}^{-}$, $M{TR}_{d}^{\pm}$, $M{TR}_{d}^{\mp}$. Visually, the images shown in the top row may be stretched vertically and horizontally, fitting the graph frames to make comparison between columns more straightforward.

### Line profile of ihMTR for various whole-brain and reduced-FOV ihMT acquisitions (CM 5B1P)


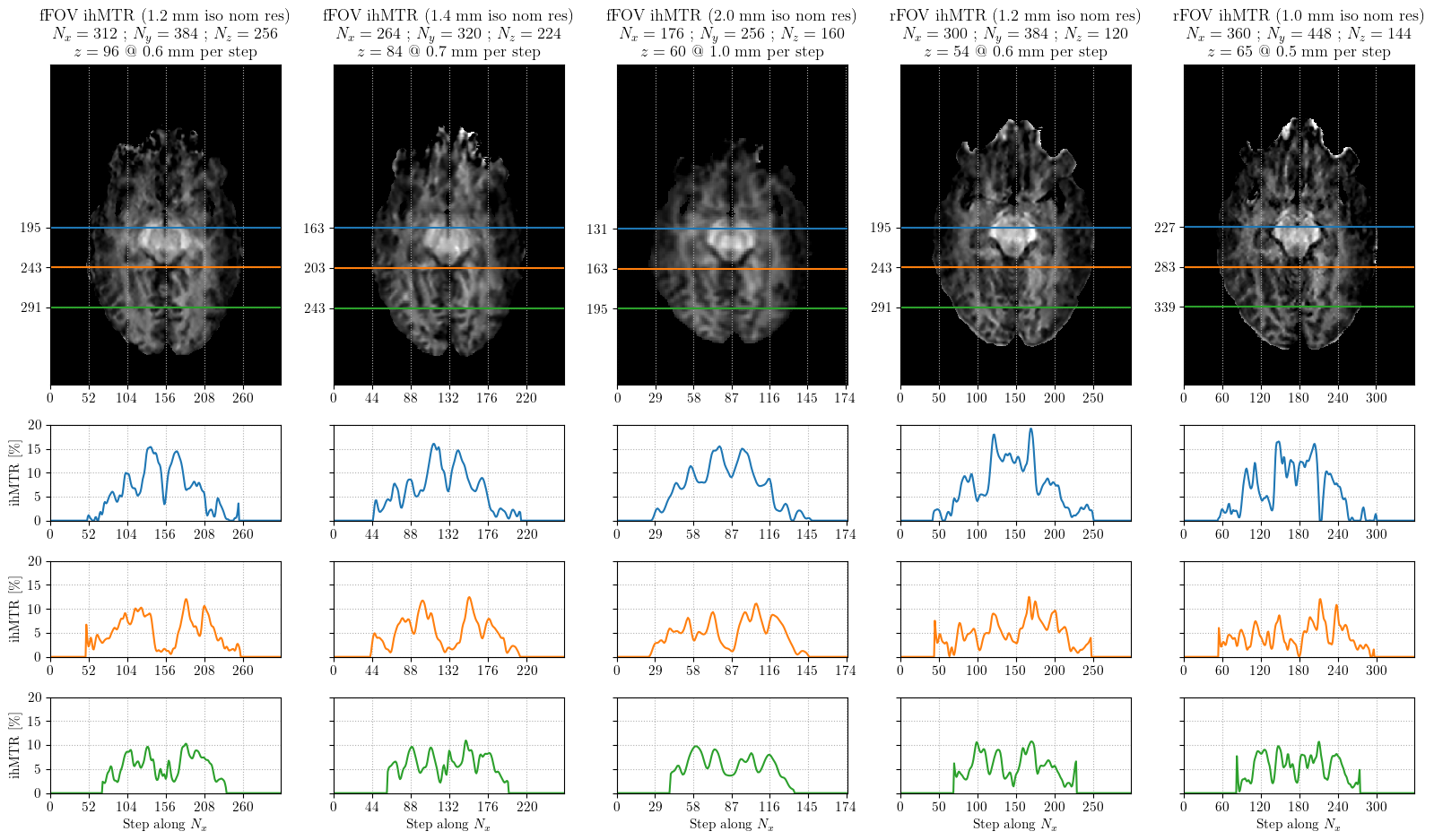


Figure S14: Line profiles of CSF-masked ihMTR images. Whole-brain are the 3 left-most columns. Reduced FOV are the 2 right-most columns. Images are not registered onto each other so that no blurring from registration occurs. Visually, the images shown in the top row may be stretched vertically and horizontally, fitting the graph frames to make comparison between columns more straightforward.

## Acquired vs advised parametrization of CM 5B1P in a whole-brain ihMT acquisition at 1.4 mm iso.


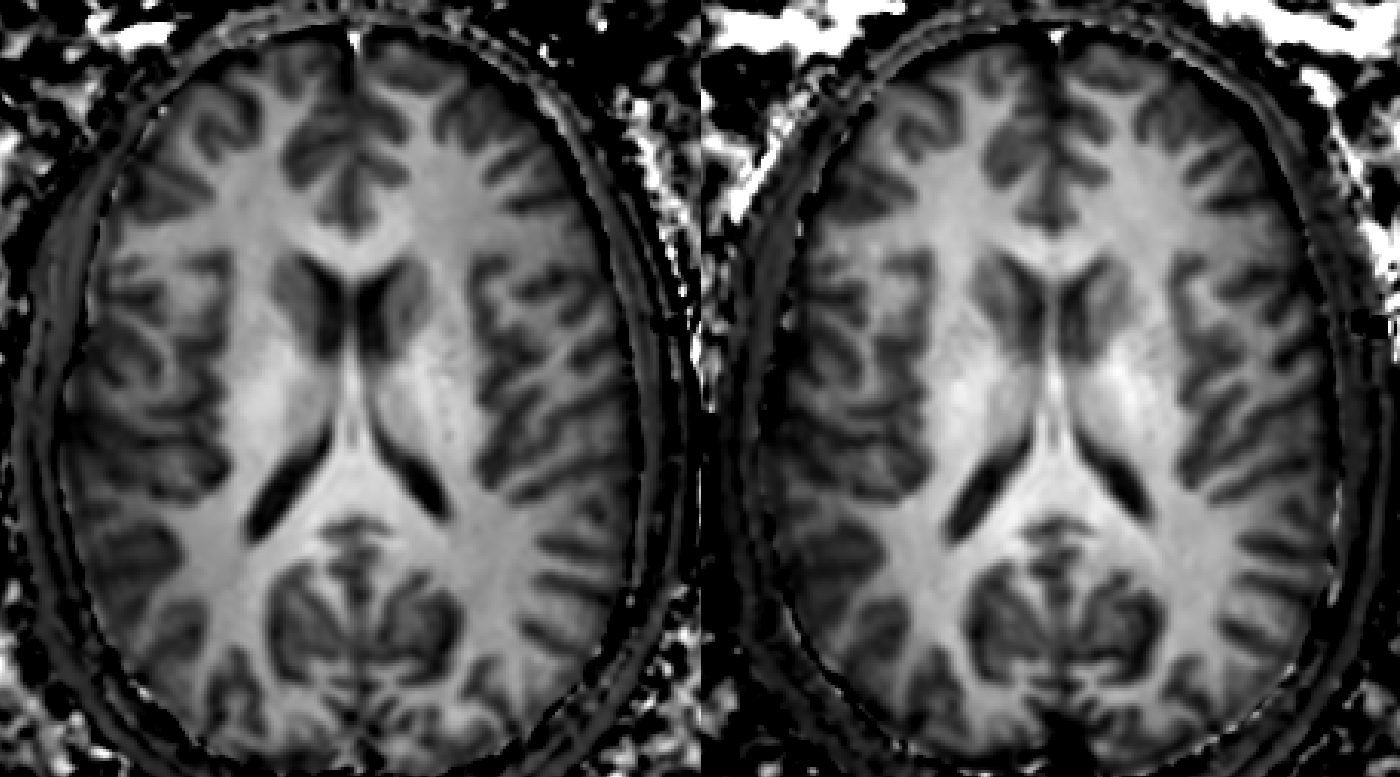


Figure S15: Representative axial slice of ihMTR images (MT preparation: cosine-modulated 5 bursts 1 pulse) with previously acquired parametrization (left) and advised parametrization (right) showing limited signal intensity differences.

## References (including references from the main document)

1. Manning AP, Chang KL, MacKay AL, Michal CA. The physical mechanism of “inhomogeneous” magnetization transfer MRI. *Journal of Magnetic Resonance*. 2017;274:125-136. doi:10.1016/j.jmr.2016.11.013

2. Varma G, Duhamel G, De Bazelaire C, Alsop DC. Magnetization transfer from inhomogeneously broadened lines: A potential marker for myelin. *Magnetic Resonance in Med*. 2015;73(2):614-622. doi:10.1002/mrm.25174

3. Varma G, Girard OM, Prevost VH, Grant AK, Duhamel G, Alsop DC. Interpretation of magnetization transfer from inhomogeneously broadened lines (ihMT) in tissues as a dipolar order effect within motion restricted molecules. *Journal of Magnetic Resonance*. 2015;260:67-76. doi:10.1016/j.jmr.2015.08.024

4. Duhamel G, Prevost VH, Cayre M, et al. Validating the sensitivity of inhomogeneous magnetization transfer (ihMT) MRI to myelin with fluorescence microscopy. *NeuroImage*. 2019;199:289-303. doi:10.1016/j.neuroimage.2019.05.061

5. Rasoanandrianina H, Grapperon AM, Taso M, et al. Region-specific impairment of the cervical spinal cord (SC) in amyotrophic lateral sclerosis: A preliminary study using SC templates and quantitative MRI (diffusion tensor imaging/inhomogeneous magnetization transfer). *NMR in Biomedicine*. 2017;30(12):e3801. doi:10.1002/nbm.3801

6. Van Obberghen E, Mchinda S, le Troter A, et al. Evaluation of the Sensitivity of Inhomogeneous Magnetization Transfer (ihMT) MRI for Multiple Sclerosis. *AJNR Am J Neuroradiol*. 2018;39(4):634-641. doi:10.3174/ajnr.A5563

7. Soustelle L, Mchinda S, Hertanu A, et al. Inhomogeneous magnetization transfer (ihMT) imaging reveals variable recovery profiles of active MS lesions according to size and localization. *Imaging Neuroscience*. 2024;2:imag-2-00235. doi:10.1162/imag_a_00235

8. Ladd ME, Bachert P, Meyerspeer M, et al. Pros and cons of ultra-high-field MRI/MRS for human application. *Progress in Nuclear Magnetic Resonance Spectroscopy*. 2018;109:1-50. doi:10.1016/j.pnmrs.2018.06.001

9. Girard OM, Prevost VH, Varma G, Cozzone PJ, Alsop DC, Duhamel G. Magnetization transfer from inhomogeneously broadened lines (ihMT): Experimental optimization of saturation parameters for human brain imaging at 1.5 Tesla: Optimizing Saturation Parameters for ihMT Brain Imaging at 1.5T. *Magn Reson Med*. 2015;73(6):2111-2121. doi:10.1002/mrm.25330

10. Panych LP, Madore B. The physics of MRI safety. *Magnetic Resonance Imaging*. 2018;47(1):28-43. doi:10.1002/jmri.25761

11. Fiedler TM, Ladd ME, Orzada S. Local and whole‐body SAR in UHF body imaging: Implications for SAR matrix compression. *Magnetic Resonance in Med*. 2025;93(2):842-849. doi:10.1002/mrm.30306

12. Bekiesińska-Figatowska M. Artifacts in Magnetic Resonance Imaging. *Pol J Radiol*. 2015;80:93-106. doi:10.12659/PJR.892628

13. Park M, Noh H, Park N. Mitigation of B1+ inhomogeneity for ultra-high-field magnetic resonance imaging: hybrid mode shaping with auxiliary EM potential. *Sci Rep*. 2020;10(1):11752. doi:10.1038/s41598-020-68651-6

14. Pohmann R, Speck O, Scheffler K. Signal‐to‐noise ratio and MR tissue parameters in human brain imaging at 3, 7, and 9.4 tesla using current receive coil arrays. *Magnetic Resonance in Med*. 2016;75(2):801-809. doi:10.1002/mrm.25677

15. Rowley CD, Campbell JSW, Wu Z, et al. A model‐based framework for correcting inhomogeneity effects in magnetization transfer saturation and inhomogeneous magnetization transfer saturation maps. *Magn Reson Med*. 2021;86(4):2192-2207. doi:10.1002/mrm.28831

16. Munsch F, Varma G, Taso M, et al. Characterization of the cortical myeloarchitecture with inhomogeneous magnetization transfer imaging (ihMT). *NeuroImage*. 2021;225:117442. doi:10.1016/j.neuroimage.2020.117442

17. Taso M, Munsch F, Girard OM, Duhamel G, Alsop DC, Varma G. Fast‐spin‐echo versus rapid gradient‐echo for 3D magnetization‐prepared acquisitions: Application to inhomogeneous magnetization transfer. *Magnetic Resonance in Med*. October 2022:mrm.29461. doi:10.1002/mrm.29461

18. Varma G, Munsch F, Burns B, et al. Three‐dimensional inhomogeneous magnetization transfer with rapid gradient‐echo (3D ihMTRAGE) imaging. *Magn Reson Med*. 2020;84(6):2964-2980. doi:10.1002/mrm.28324

19. Rowley CD, Campbell JSW, Leppert IR, Nelson MC, Pike GB, Tardif CL. Optimization of acquisition parameters for cortical inhomogeneous magnetization transfer (ihMT) imaging using a rapid gradient echo readout. *Magnetic Resonance in Med*. 2023;90(5):1762-1775. doi:10.1002/mrm.29754

20. Marques JP, Kober T, Krueger G, van der Zwaag W, Van de Moortele PF, Gruetter R. MP2RAGE, a self bias-field corrected sequence for improved segmentation and T1-mapping at high field. *NeuroImage*. 2010;49(2):1271-1281. doi:10.1016/j.neuroimage.2009.10.002

21. Massire A, Seiler C, Troalen T, et al. T1-Based Synthetic Magnetic Resonance Contrasts Improve Multiple Sclerosis and Focal Epilepsy Imaging at 7 T. *Invest Radiol*. 2021;56(2):127-133. doi:10.1097/RLI.0000000000000718

22. Chung S, Kim D, Breton E, Axel L. Rapid *B*_1_^+^ mapping using a preconditioning RF pulse with TurboFLASH readout. *Magnetic Resonance in Med*. 2010;64(2):439-446. doi:10.1002/mrm.22423

23. Mchinda S, Varma G, Prevost VH, et al. Whole brain inhomogeneous magnetization transfer (ihMT) imaging: Sensitivity enhancement within a steady‐state gradient echo sequence. *Magnetic Resonance in Med*. 2018;79(5):2607-2619. doi:10.1002/mrm.26907

24. Soustelle L, Troalen T, Hertanu A, et al. A strategy to reduce the sensitivity of inhomogeneous magnetization transfer (ihMT) imaging to radiofrequency transmit field variations at 3 T. *Magnetic Resonance in Med*. 2022;87(3):1346-1359. doi:10.1002/mrm.29055

25. Harris FJ. On the use of windows for harmonic analysis with the discrete Fourier transform. *Proc IEEE*. 1978;66(1):51-83. doi:10.1109/PROC.1978.10837

26. Alsop DC, Ercan E, Girard OM, et al. Inhomogeneous magnetization transfer imaging: Concepts and directions for further development. *NMR in Biomedicine*. August 2022. doi:10.1002/nbm.4808

27. Marques JP, Gruetter R. New Developments and Applications of the MP2RAGE Sequence - Focusing the Contrast and High Spatial Resolution R1 Mapping. Yacoub E, ed. *PLoS ONE*. 2013;8(7):e69294. doi:10.1371/journal.pone.0069294

28. Collins DL, Zijdenbos AP, Baaré WFC, Evans AC. ANIMAL+INSECT: Improved Cortical Structure Segmentation. In: Kuba A, Šáamal M, Todd-Pokropek A, eds. *Information Processing in Medical Imaging*. Vol 1613. Lecture Notes in Computer Science. Berlin, Heidelberg: Springer Berlin Heidelberg; 1999:210-223. doi:10.1007/3-540-48714-X_16

29. Soustelle L, Lamy J, Le Troter A, et al. *A Motion Correction Strategy for Multi-Contrast Based 3D Parametric Imaging: Application to Inhomogeneous Magnetization Transfer (ihMT)*. bioRxiv; 2020. doi:10.1101/2020.09.11.292649

30. Henkelman RM, Stanisz GJ, Graham SJ. Magnetization transfer in MRI: a review. *NMR in Biomedicine*. 2001;14(2):57-64. doi:10.1002/nbm.683

31. Johnson NL, Kotz S, Balakrishnan N. Continuous univariate distributions. 2. In: 2. ed. New York: Wiley; 1995.

32. Müller DK, Pampel A, Möller HE. Matrix-algebra-based calculations of the time evolution of the binary spin-bath model for magnetization transfer. *Journal of Magnetic Resonance*. 2013;230:88-97. doi:10.1016/j.jmr.2013.01.013

33. Fritsch FN, Butland J. A Method for Constructing Local Monotone Piecewise Cubic Interpolants. *SIAM J Sci and Stat Comput*. 1984;5(2):300-304. doi:10.1137/0905021

34. Holtzman WH. The Unbiased Estimate of the Population Variance and Standard Deviation. *The American Journal of Psychology*. 1950;63(4):615. doi:10.2307/1418879

35. Stacy EW. A Generalization of the Gamma Distribution. *Ann Math Statist*. 1962;33(3):1187-1192. doi:10.1214/aoms/1177704481

36. Aja‐Fernández S, Tristán‐Vega A, Hoge WS. Statistical noise analysis in GRAPPA using a parametrized noncentral Chi approximation model. *Magnetic Resonance in Med*. 2011;65(4):1195-1206. doi:10.1002/mrm.22701

37. Bushberg J, Seibert JA, Leidholdt EM, Boone JM. *The Essential Physics of Medical Imaging*. 4th edition. Philadelphia: Wolters Kluwer Medical; 2021.

38. Min K, Sohn B, Kim WJ, et al. A human brain atlas of *χ* ‐separation for normative iron and myelin distributions. *NMR in Biomedicine*. 2024;37(12):e5226. doi:10.1002/nbm.5226

39. Lam MH, Novoselova M, Yung A, et al. Interpretation of inhomogeneous magnetization transfer in myelin water using a four‐pool model with dipolar reservoirs. *Magnetic Resonance in Med*. 2025;94(1):278-292. doi:10.1002/mrm.30465

40. Wallstein N, Pampel A, Müller R, Jäger C, Morawski M, Möller HE. An unconstrained four pool model analysis of proton relaxation and magnetization transfer in ex vivo white matter. *Sci Rep*. 2025;15(1):4354. doi:10.1038/s41598-025-87362-4

41. Pohmann R, Scheffler K. A theoretical and experimental comparison of different techniques for *B*_1_ mapping at very high fields. *NMR in Biomedicine*. 2013;26(3):265-275. doi:10.1002/nbm.2844

42. Deniz CM. Parallel Transmission for Ultrahigh Field MRI. *Topics in Magnetic Resonance Imaging*. 2019;28(3):159-171. doi:10.1097/RMR.0000000000000204

43. Leitão D, Tomi‐Tricot R, Bridgen P, et al. Parallel transmit pulse design for saturation homogeneity (PUSH) for magnetization transfer imaging at 7T. *Magnetic Resonance in Med*. 2022;88(1):180-194. doi:10.1002/mrm.29199

44. Brown RW, Cheng YCN, Haacke EM, Thompson MR, Venkatesan R. *Magnetic Resonance Imaging: Physical Principles and Sequence Design*. Second edition. Hoboken, New Jersey: John Wiley & Sons, Inc; 2014.

45. Mezrich R. A perspective on K-space. *Radiology*. 1995;195(2):297-315. doi:10.1148/radiology.195.2.7724743

46. ISO. *ISO IEC 14882:2017 Information Technology — Programming Languages — C++*. Fifth. pub-ISO:adr: pub-ISO; 2017. https://www.iso.org/standard/68564.html.

47. Schäling B. *The Boost C++ Libraries: Introduces 72 Libraries with More than 430 Examples*. 2nd English ed. Laguna Hills, Calif: XML Press; 2014.

48. Tustison NJ, Cook PA, Holbrook AJ, et al. The ANTsX ecosystem for quantitative biological and medical imaging. *Sci Rep*. 2021;11(1):9068. doi:10.1038/s41598-021-87564-6

49. Tustison NJ, Yassa MA, Rizvi B, et al. ANTsX neuroimaging-derived structural phenotypes of UK Biobank. *Sci Rep*. 2024;14(1):8848. doi:10.1038/s41598-024-59440-6

50. Yoo Terry S., Ackerman Michael J., Lorensen William E., et al. Engineering and Algorithm Design for an Image Processing API: A Technical Report on ITK - the Insight Toolkit. In: *Studies in Health Technology and Informatics*. IOS Press; 2002. doi:10.3233/978-1-60750-929-5-586

51. McCormick M, Liu X, Jomier J, Marion C, Ibanez L. ITK: enabling reproducible research and open science. *Front Neuroinform*. 2014;8. doi:10.3389/fninf.2014.00013

52. Avants BB, Tustison NJ, Song G, Cook PA, Klein A, Gee JC. A reproducible evaluation of ANTs similarity metric performance in brain image registration. *NeuroImage*. 2011;54(3):2033-2044. doi:10.1016/j.neuroimage.2010.09.025

53. Avants B, Epstein C, Grossman M, Gee J. Symmetric diffeomorphic image registration with cross-correlation: Evaluating automated labeling of elderly and neurodegenerative brain. *Medical Image Analysis*. 2008;12(1):26-41. doi:10.1016/j.media.2007.06.004

54. Klein A, Andersson J, Ardekani BA, et al. Evaluation of 14 nonlinear deformation algorithms applied to human brain MRI registration. *NeuroImage*. 2009;46(3):786-802. doi:10.1016/j.neuroimage.2008.12.037

55. Fischl B. FreeSurfer. *NeuroImage*. 2012;62(2):774-781. doi:10.1016/j.neuroimage.2012.01.021

56. Hoopes A, Mora JS, Dalca AV, Fischl B, Hoffmann M. SynthStrip: skull-stripping for any brain image. *NeuroImage*. 2022;260:119474. doi:10.1016/j.neuroimage.2022.119474

57. Jenkinson M, Beckmann CF, Behrens TEJ, Woolrich MW, Smith SM. FSL. *NeuroImage*. 2012;62(2):782-790. doi:10.1016/j.neuroimage.2011.09.015

58. Billot B, Greve DN, Puonti O, et al. SynthSeg: Segmentation of brain MRI scans of any contrast and resolution without retraining. *Medical Image Analysis*. 2023;86:102789. doi:10.1016/j.media.2023.102789

59. Billot B, Magdamo C, Cheng Y, Arnold SE, Das S, Iglesias JE. Robust machine learning segmentation for large-scale analysis of heterogeneous clinical brain MRI datasets. *Proc Natl Acad Sci USA*. 2023;120(9):e2216399120. doi:10.1073/pnas.2216399120

60. Fonov V, Evans A, McKinstry R, Almli C, Collins D. Unbiased nonlinear average age-appropriate brain templates from birth to adulthood. *NeuroImage*. 2009;47:S102. doi:10.1016/S1053-8119(09)70884-5

61. Fonov V, Evans AC, Botteron K, Almli CR, McKinstry RC, Collins DL. Unbiased average age-appropriate atlases for pediatric studies. *NeuroImage*. 2011;54(1):313-327. doi:10.1016/j.neuroimage.2010.07.033

62. Anaconda. Anaconda Software Distribution. November 2016. https://anaconda.com.

63. conda contributors. conda: A system-level, binary package and environment manager running on all major operating systems and platforms. March 2023. https://docs.conda.io/projects/conda/.

64. Rossum G van, Drake FL. *The Python Language Reference*. Release 3.0.1 [Repr.]. Hampton, NH: Python Software Foundation; 2010.

65. Tournier JD, Smith R, Raffelt D, et al. MRtrix3: A fast, flexible and open software framework for medical image processing and visualisation. *NeuroImage*. 2019;202:116137. doi:10.1016/j.neuroimage.2019.116137

66. Veraart J, Fieremans E, Novikov DS. Diffusion MRI noise mapping using random matrix theory. *Magnetic Resonance in Med*. 2016;76(5):1582-1593. doi:10.1002/mrm.26059

67. Veraart J, Novikov DS, Christiaens D, Ades-aron B, Sijbers J, Fieremans E. Denoising of diffusion MRI using random matrix theory. *NeuroImage*. 2016;142:394-406. doi:10.1016/j.neuroimage.2016.08.016

68. Cordero-Grande L, Christiaens D, Hutter J, Price AN, Hajnal JV. Complex diffusion-weighted image estimation via matrix recovery under general noise models. *NeuroImage*. 2019;200:391-404. doi:10.1016/j.neuroimage.2019.06.039

69. Virtanen P, Gommers R, Oliphant TE, et al. SciPy 1.0: fundamental algorithms for scientific computing in Python. *Nat Methods*. 2020;17(3):261-272. doi:10.1038/s41592-019-0686-2

70. Yushkevich PA, Piven J, Hazlett HC, et al. User-guided 3D active contour segmentation of anatomical structures: Significantly improved efficiency and reliability. *NeuroImage*. 2006;31(3):1116-1128. doi:10.1016/j.neuroimage.2006.01.015

71. Hunter JD. Matplotlib: A 2D Graphics Environment. *Comput Sci Eng*. 2007;9(3):90-95. doi:10.1109/MCSE.2007.55

72. The Matplotlib Development Team. Matplotlib: Visualization with Python. July 2025. doi:10.5281/ZENODO.16644850

73. Henkelman RM, Huang X, Xiang Q, Stanisz GJ, Swanson SD, Bronskill MJ. Quantitative interpretation of magnetization transfer. *Magnetic Resonance in Med*. 1993;29(6):759-766. doi:10.1002/mrm.1910290607

74. Morrison C, Mark Henkelman R. A Model for Magnetization Transfer in Tissues. *Magn Reson Med*. 1995;33(4):475-482. doi:10.1002/mrm.1910330404

75. Morrison C, Stanisz G, Henkelman RM. Modeling Magnetization Transfer for Biological-like Systems Using a Semi-solid Pool with a Super-Lorentzian Lineshape and Dipolar Reservoir. *Journal of Magnetic Resonance, Series B*. 1995;108(2):103-113. doi:10.1006/jmrb.1995.1111

76. Provotorov BN. Magnetic Resonance Saturation in Crystals. Soviet Physics Jetp-Ussr, 1962, vol. 14, no 5, p. 1126-1131

77. The MathWorks Inc. MATLAB. September 2024. https://www.mathworks.com.

78. McConnell HM. Reaction Rates by Nuclear Magnetic Resonance. *The Journal of Chemical Physics*. 1958;28(3):430-431. doi:10.1063/1.1744152

79. Wallstein N, Pampel A, Jäger C, Müller R, Möller HE. Anisotropic longitudinal water proton relaxation in white matter investigated ex vivo in porcine spinal cord with sample rotation. *Sci Rep*. 2024;14(1):12961. doi:10.1038/s41598-024-63483-0

80. Pampel A, Müller DK, Anwander A, Marschner H, Möller HE. Orientation dependence of magnetization transfer parameters in human white matter. *NeuroImage*. 2015;114:136-146. doi:10.1016/j.neuroimage.2015.03.068

81. Graham SJ, Henkelman RM. Understanding pulsed magnetization transfer. *Magnetic Resonance Imaging*. 1997;7(5):903-912. doi:10.1002/jmri.1880070520

82. Harris CR, Millman KJ, Van Der Walt SJ, et al. Array programming with NumPy. *Nature*. 2020;585(7825):357-362. doi:10.1038/s41586-020-2649-2

83. Waskom M. seaborn: statistical data visualization. *JOSS*. 2021;6(60):3021. doi:10.21105/joss.03021

84. Billingsley P. *Probability and Measure*. Hoboken, N.J.: Wiley; 2013.
